# Tailoring Vascular‐Immune Homeostasis via Manganese‐DNA Complex‐Armed Immunogenic Extracellular Vesicles for Pancreatic Cancer Immunotherapy

**DOI:** 10.1002/advs.202507159

**Published:** 2025-10-22

**Authors:** Xue Jiang, Lihuan Shang, Xiaochun Chen, Xiangzhan Kong, Xiaojuan Wang, Linjia Jiang, Huiying Zhao, Yafeng Zhu, Cheng Huang, Shiyi Deng, Rui Zhang, Minghui Wang, Haoming Lin, Ping‐Pui Wong

**Affiliations:** ^1^ Guangdong Provincial Key Laboratory of Malignant Tumor Epigenetics and Gene Regulation Guangdong‐Hong Kong Joint Laboratory for RNA medicine Sun Yat‐sen Memorial Hospital State Key Laboratory of Oncology in South China Sun Yat‐sen University Guangzhou 510120 China; ^2^ Medical Research Center Sun Yat‐sen Memorial Hospital Sun Yat‐sen University Guangzhou 510120 China; ^3^ Guangzhou Key Laboratory of Precise Diagnosis and Treatment of Biliary Tract Cancer Department of Biliary‐Pancreatic Surgery Sun Yat‐sen Memorial Hospital Sun Yat‐sen University Guangzhou 510120 China; ^4^ Department of Thoracic surgery Sun Yat‐sen Memorial Hospital Sun Yat‐sen University Guangzhou 510120 China

**Keywords:** cGAS‐STING, dendritic cells, immunotherapy, manganese‐DNA complex, pancreatic cancer

## Abstract

Harnessing the cGAS‐STING DNA sensing pathway in dendritic cells (DCs) to enhance anti‐tumor immunity in immunosuppressive pancreatic tumors remains a significant challenge. While manganese (Mn^2+^) enhances cGAS sensitivity to DNA, the precise mechanisms and potential of Mn‐DNA complexes in this process are unclear. Here, this work introduces a strategy to encapsulate Mn‐DNA complexes into DC‐derived immunogenic extracellular vesicle (EV_DC_@Mn‐DNA) to trigger robust anti‐tumor immunity. This work shows that Mn^2+^ induces tumor cell‐DNA transition to Z‐DNA, strengthening cGAS binding and promoting its phase condensation for optimal activation in DCs. Using this newly developed Raft‐Ultra method, this work engineers immunogenic EVs derived from tumor cell lysate‐pulsed DCs, loaded with Mn‐DNA complexes (EV_DC_@Mn‐DNA). These EVs efficiently deliver Mn‐DNA complexes to DCs, activating the cGAS‐STING pathway both in vitro and in vivo. In animal models, EV_DC_@Mn‐DNA administration enhances vascular function, as evidenced by increased blood flow and perfusion, improved anti‐PD‐L1 delivery, reduced hypoxia, and elevated endothelial cell‐ICAM1 expression, which facilitates T cell adhesion. This approach expands the intratumoral population of activated DCs and T cells and promotes the formation of larger tertiary lymphoid structures, ultimately suppressing orthotopic pancreatic tumor growth. Overall, this EV_DC_@Mn‐DNA strategy reprograms intratumoral DCs, restores vascular‐immune homeostasis, and potentiates anti‐tumor immunity in pancreatic cancer.

## Graphical abstract

This study demonstrates that Mn^2^⁺–tumor DNA complexes encapsulated in dendritic cell (DC)– derived immunogenic extracellular vesicles (EV_DC_@Mn‐DNA) act as a DC‐specific cGAS– STING activator. EV_DC_@Mn‐DNA treatment enhances intratumoral DC activation, improves tumor vascular function, promotes CD8⁺ T cell activity, and suppresses pancreatic tumor growth, highlighting a DC‐targeted, metal ion–based immunotherapy.

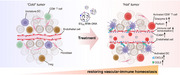

## 1 Introduction

Immunotherapy has been researched extensively for pancreatic ductal adenocarcinoma (PDAC), the most prevalent type of pancreatic cancer. However, challenges persist due to abnormal vasculature, leading to inadequate immune cell infiltration and the development of an immunosuppressive tumor microenvironment.^[^ ^1^, ^2^, ^3^ ^]^ Aberrant tumor vascular functions lead to insufficient blood flow/perfusion and downregulation of adhesion molecule expression, hindering intratumor immune cell infiltration.^[^ ^4^, ^5^ ^]^ Efforts currently underway to develop therapies for remodeling tumor vasculature are showing promise in reshaping the tumor microenvironment.^[^ ^6^, ^7^ ^]^ However, further improvements are needed to enhance its efficacy in transforming immune‐deserted pancreatic tumors into an immune‐inflamed phenotype.

The cyclic guanosine monophosphate–adenosine monophosphate synthase–stimulator of interferon genes (cGAS–STING) DNA‐sensing pathway, which signals through downstream effectors TANK‐binding kinase 1 (TBK1) and interferon regulatory factor 3 (IRF3), has emerged as a key target in cancer therapy, orchestrating both innate and adaptive immune responses.^[^ ^8^, ^9^ ^]^ Conventional STING agonists, which induce cytokines such as type I interferons (IFNs), can enhance dendritic cell (DC) antigen presentation and promote cytotoxic CD8⁺ T cell activity.^[^ ^10^, ^11^ ^]^ Notably, Wang‐Bishop et al. found that STING agonists could mediate tumor blood vessel function, fostering immune cell infiltration into the tumor parenchyma and triggering a robust systemic anti‐tumor immune response.^[^ ^12^ ^]^ Interestingly, activated DCs have been implicated in tumor vasculature modulation,^[^ ^13^ ^]^ though further evidence is required. Despite these promising findings in animal models, the clinical efficacy of STING agonists has been limited, probably attributed to their poor pharmacokinetic and physicochemical properties, low efficacy targeting DCs within tumors, adverse effect arising from systemic delivery and random diffusion, which can lead to unwanted autoimmune toxicity, necessitating intra‐tumoral dosing.^[^ ^14^, ^15^ ^]^ In contrast, tumor‐derived DNA may represent a promising alternative. It can naturally activate the cGAS–STING pathway in a length‐dependent but sequence‐independent manner,^[^ ^8^, ^16^ ^]^ potentially bypassing many limitations of synthetic STING agonists. Notably, DNA can adopt different structural isoforms, most commonly right‐handed B‐DNA, whereas left‐handed Z‐DNA forms under specific conditions.^[^ ^17^ ^]^ These isoforms may affect cGAS binding and its liquid‐phase condensation, a process involved in regulating cGAS–STING activation and the resulting immune response.^[^ ^18^ ^]^ To evaluate this, in vitro phase condensation assays were performed by mixing purified cGAS protein and DNA under molecular crowding conditions to monitor cGAS condensate droplet formation.^[^ ^19^ ^]^ However, it remains unclear which isoform—B‐DNA or Z‐DNA—optimally promotes cGAS‐mediated immune activation. Developing a method to package tumor DNA, protect it from degradation, and deliver it to DCs may provide an effective way to engage cGAS–STING signaling for cancer immunotherapy.

Extracellular vesicles (EVs), including microvesicles, are lipid membrane‐bound vesicles that have been engineered for delivering drugs and DNA.^[^ ^20^, ^21^, ^22^ ^]^ Recent studies suggest that mature dendritic cells (DCs) can be stimulated to secrete immunogenic EVs, including microvesicles, which are capable of delivering cargo to intratumoral DCs,^[^ ^20^, ^23^ ^]^ suggesting their potential for targeted DNA delivery to DCs. Additionally, metal ions like manganese (Mn^2+^) have been shown to regulate cGAS sensitivity to DNA and participate in cGAS‐STING pathway activation,^[^ ^24^, ^25^, ^26^, ^27^, ^28^ ^]^ although the precise mechanism remains unclear. It is not yet known whether coordination of metal ions such as Mn^2^⁺ with DNA can alter DNA conformation and enhance its binding to cGAS. Therefore, packaging Mn–DNA complexes into DC‐derived EVs may offer a potential strategy to selectively engage the cGAS–STING pathway in intratumoral DCs.

In this study, we developed a cell type‐specific cGAS‐targeting strategy by coordinating Mn^2^⁺ with tumor cell‐derived DNA to form Mn‐DNA complexes, which were subsequently encapsulated into DC‐derived immunogenic EVs (EV_DC_@Mn‐DNA). Administration of EV_DC_@Mn‐DNA effectively activated cGAS–STING signaling in DCs, resulting in enhanced anti‐tumor immunity and suppression of orthotopic pancreatic tumor growth. These findings highlight the advantage of DC‐targeted cGAS‐STING activation over other STING agonists, which lack cell type specificity, as a more precise and effective metal ion‐based therapeutic approach for pancreatic cancer.

## 2 Results

### 2.1 Mn^2^⁺ Interacts with DNA to Modify its Conformation, Strengthening cGAS Binding and Promoting cGAS Phase Condensation, Thereby Optimizing cGAS‐STING‐TBK1 Pathway Activation in DCs

**Figure 1:**
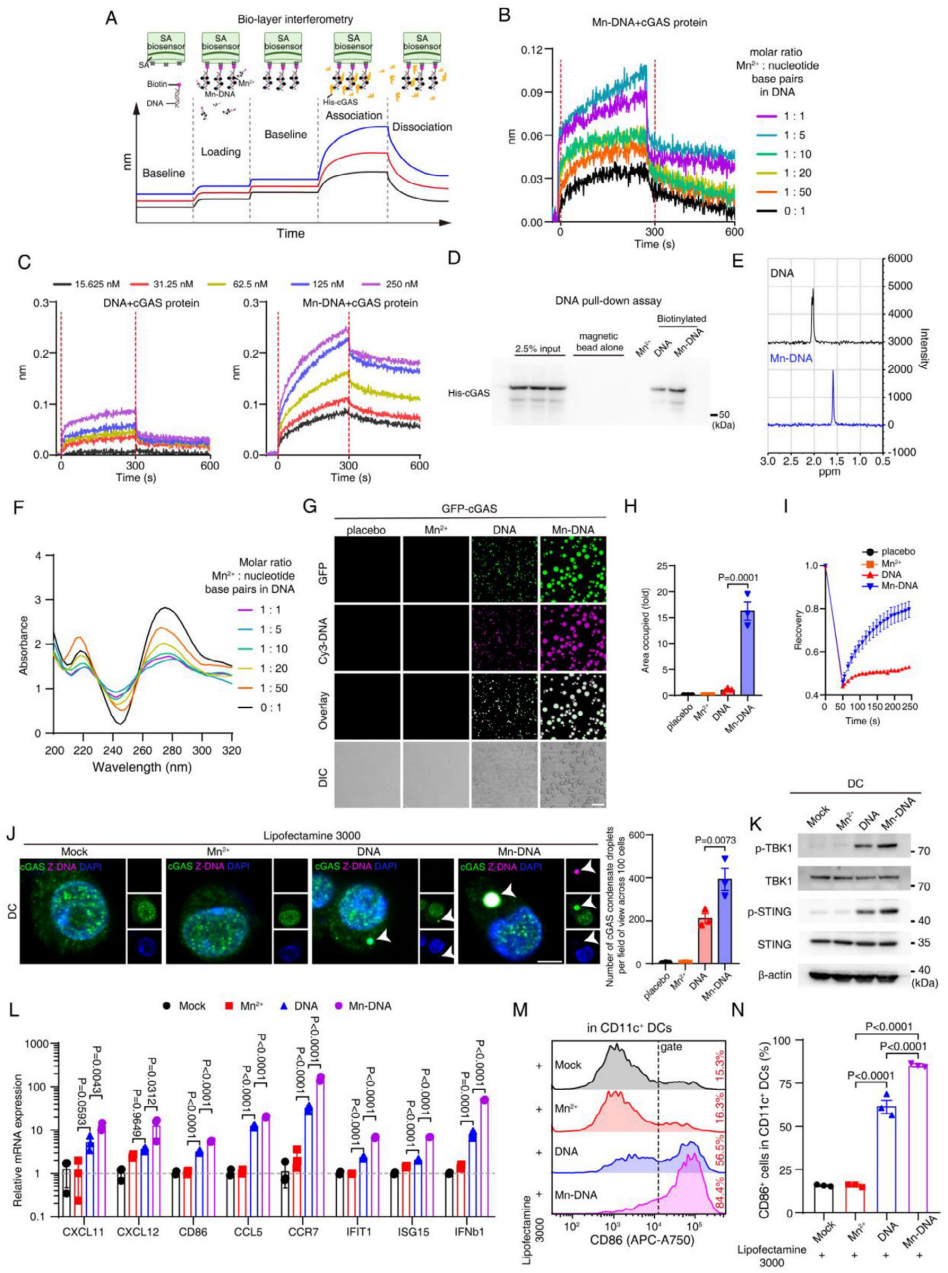
Mn^2^⁺ alters the conformation of tumor cell‐derived DNA to enhance its cGAS binding and promote cGAS phase separation, thereby maximizing activation of the cGAS‐STING pathway. A) The schematic diagram illustrates the workflow of the biolayer interferometry (BLI) assay. Streptavidin (SA)‐coated biosensors were first equilibrated in buffer and then incubated with biotinylated Mn‐DNA to allow immobilization. After a subsequent equilibration step in buffer, the probes were incubated with recombinant cGAS protein to measure the binding and dissociation kinetics between Mn‐DNA and cGAS. The different colored lines represent the various experimental groups, shown here as an example. B) DNA‐protein interaction assays were conducted to measure the kinetic dynamics of binding affinity between Mn‐DNA complexes with increasing molar ratios of Mn^2+^ to nucleotide base pairs in DNA and purified His‐cGAS protein. C) DNA‐protein interaction assays were performed using DNA alone at various concentrations and Mn‐DNA complexes (at a 1:5 molar ratio) with DNA at different concentrations, along with purified His‐cGAS protein. D) Western blot analysis of DNA pull‐down assays demonstrating the binding affinity of purified cGAS protein with biotinylated DNA/Mn‐DNA complexes or Mn^2+^. A 2.5% input was loaded onto the gel, with magnetic beads serving as a negative control. E) NMR phosphorus spectroscopy analysis of DNA and Mn‐DNA complexes. F) Circular dichroism spectrum analysis of Mn‐DNA complexes at various Mn^2^⁺ molar ratios to nucleotide base pairs in DNA. G) In vitro phase condensation experiment of Cys3‐labeled Mn‐DNA complexes and GFP‐cGAS protein. Representative images for each treatment group are shown. H) Bar chart depicting changes in the phase separation droplet area of GFP‐cGAS protein under different conditions. I) Graph depicting fluorescence intensity recovery following photobleaching of phase separation droplets formed by GFP‐cGAS protein under different treatments. J) Representative fluorescent images of cGAS, Z‐DNA and DAPI staining in DCs post‐treatment. Merged and individual fluorescent channel images are shown. White arrows highlight cGAS phase condensation. Bar chart depicting the number of cGAS condensate droplets per field of view across 100 cells (n = 3 independent experiments). K) Western blot analysis of mouse DCs with indicated proteins after treatment. L) Mouse DCs were transfected with either Mock, Mn^2^⁺, DNA, or Mn‐DNA complexes using Lipofectamine 3000 transfection reagent. Bar chart shows relative mRNA expression of specified genes in DCs after treatment (n = 3 independent experiments). M) Flow cytometry analysis of DC maturation marker CD86 expression in CD11c^+^ DCs after treatment. N) Bar chart showing the percentage of CD86^+^ DCs in the total CD11c^+^ DC population in each group (n = 3 independent experiments). Results represent mean ± S.E.M. H, J, N) One‐way ANOVA with Tukey's post hoc test. L) Two‐way ANOVA with Tukey's post hoc test. Scale bars in (G) represent 10 µm. J) 5 µm.

Manganese (Mn^2+^) has emerged as a significant player in cancer immunotherapy, acting as a potential STING agonist or co‐activator.^[^ ^25^ ^]^ Recent research suggests that Mn^2+^ not only triggers cGAS to produce cGAMP and activate the type I interferon signaling pathway but also increases cGAS sensitivity to DNA.^[^ ^26^, ^29^ ^]^ However, the precise mechanism by which Mn^2+^ achieves this remains unclear. We therefore hypothesized that Mn^2+^ could boost DNA binding to cGAS. To investigate, Mn–DNA complexes were prepared by mixing Mn^2^⁺ at different molar ratios with fragmented genomic DNA (200–1000 bp) derived from DT6066 mouse pancreatic tumor cells, a commonly used model for both in vitro and in vivo studies^[^ ^6^, ^30^ ^]^ (Figure S1A, Supporting Information). We measured the manganese content in Mn‐DNA complexes using inductively coupled plasma atomic emission spectroscopy (ICP‐AES), which was used to assess the molecular ratio of Mn^2^⁺ to nucleotide base pairs, confirming Mn^2^⁺ incorporation into DNA at the specified ratios (Figure S1B, Supporting Information). We then assessed their binding affinity with cGAS using bio‐layer interferometry, as depicted in the schematic diagram (**Figure** **1**A). Significantly, enhancing the molar ratio of Mn^2+^ to nucleotide base pairs in DNA resulted in improved binding affinity of Mn‐DNA complexes for cGAS, with the optimal affinity noted at a 1:5 molar ratio of Mn^2+^ to nucleotide base pairs in DNA (Figure 1B). Further DNA binding assays revealed that the inclusion of Mn^2+^ enhanced DNA binding to cGAS across various DNA concentrations, surpassing the binding observed with DNA alone (Figure 1C). Subsequently, pull‐down experiments showed Mn‐DNA complexes pulled down more cGAS protein than DNA alone (Figure 1D). To investigate the reason behind the enhanced affinity of Mn‐DNA complexes for cGAS, we analyzed the conformation of Mn‐DNA complex and DNA alone using nuclear magnetic resonance phosphorus spectroscopy. The results showed a significant chemical shift of phosphorus signals in the Mn‐DNA complex toward the high‐field region as compared to DNA alone (Figure 1E), suggesting that DNA conformation may have changed upon coordination with Mn^2^⁺. Subsequently, we further analyzed the conformation of Mn‐DNA using circular dichroism spectroscopy. The results indicated that as the Mn^2^⁺ ratio in the Mn‐DNA complex increased, its right‐handed optical rotation gradually weakened (Figure 1F). Given that previous studies have shown Z‐DNA to exhibit left‐handed optical rotation,^[^ ^31^ ^]^ this suggests that the proportion of Mn‐DNA adopting a Z‐DNA conformation increased with higher Mn^2^⁺ content, thereby neutralizing the overall right‐handed optical rotation of the DNA fragment (Figure 1F). To further verify the affinity between Mn‐DNA complexes and cGAS, we conducted in vitro phase condensation experiments. Compared to DNA alone, Mn‐DNA complexes formed larger phase‐separated droplets with cGAS, as evidenced by a substantial increase in droplet area in the field of view, with the optimal phase condensation noted at a 1:5 molar ratio of Mn^2+^ to nucleotide base pairs in DNA (Figure 1G,H and Figure S1C, Supporting Information). However, the addition of placebo or Mn^2+^ alone had no effect on inducing cGAS phase separation (Figure 1G,H). Additionally, fluorescence recovery after photobleaching experiments demonstrated that the fluorescence signal in the bleached region of Mn‐DNA/cGAS phase‐separated droplets gradually recovered (Figure 1I), confirming the dynamic fluidity of these phase‐separated structures. Importantly, we demonstrated that among various metal ions, including Calcium (Ca^2^⁺), Aluminum (Al^3^⁺), Iron (Fe^2^⁺), Magnesium (Mg^2^⁺), Zinc (Zn^2^⁺), and Mn^2^⁺, only Mn‐DNA complexes induced a greater degree of cGAS phase separation as compared to DNA complexes formed with other metal ions (Figure S1D, Supporting Information). Consistent with previous studies, which indicate that DNA binding to cGAS induced the formation of lipid‐like droplets in which cGAS was activated,^[^ ^18^ ^]^ our findings highlight the potential advantage of utilizing Mn‐DNA complexes to effectively target cGAS phase condensation and modulate this pathway activity.

To assess whether Mn‐DNA complexes induce intracellular cGAS phase separation, we transfected mouse bone marrow‐derived dendritic cells with either DNA alone or Mn‐DNA complexes using Lipofectamine 3000. Cells were then subjected to cGAS, Z‐DNA, and DAPI staining, followed by Western blot analysis. Mn‐DNA transfection induced cGAS phase separation, as shown by cGAS condensation into droplet‐like structures co‐localized with Z‐DNA, whereas DCs transfected with DNA alone showed no Z‐DNA staining. Notably, Mn‐DNA complexes formed larger cGAS condensates and increased the number of cGAS condensate droplets compared with DNA alone, while placebo or Mn^2^⁺ alone failed to trigger cGAS phase separation (Figure 1J). Western blot analysis further revealed that activation of the cGAS‐STING pathway, as indicated by increased phosphorylation of STING and its downstream target TBK1, was highest in DCs transfected with Mn‐DNA complexes compared to those transfected with DNA alone or treated with mock or Mn^2+^ alone (Figure 1K). Furthermore, to assess the ability of Mn‐DNA complexes to stimulate the cGAS–STING pathway in the mouse DC cell line DC2.4—a widely used model for studying DC function^[^ ^20^, ^32^ ^]^—we transfected Mn‐DNA complexes into the cells using Lipofectamine 3000 transfection reagent. RT‐PCR results showed Mn‐DNA complexes more strongly activated the molecules involved in type I interferon (cGAS‐STING) pathway as well as DC maturation and T cell recruitment, such as CXCL11, CXCL12, CD86, CCL5, CCR7, IFIT1 and ISG15 and IFNb1, compared to DNA alone, while Mn^2+^ alone had minimal effect (Figure 1L). Additionally, flow cytometry (FACS) revealed Mn‐DNA complexes more effectively induced mouse bone marrow‐derived dendritic cell maturation than DNA alone (Figure 1M,N). Overall, our results demonstrate that Mn^2+^ enhances DNA binding to cGAS and promote its phase condensation, thereby amplifying cGAS‐STING pathway activation and facilitating DC maturation. These findings support the potential of manganese‐based cGAS‐STING pathway activators and warrant further investigation.

### 2.2 Preparation and Characterization DC‐Derived Extracellular Vesicles Encapsulating Mn‐DNA Complexes

**Figure 2:**
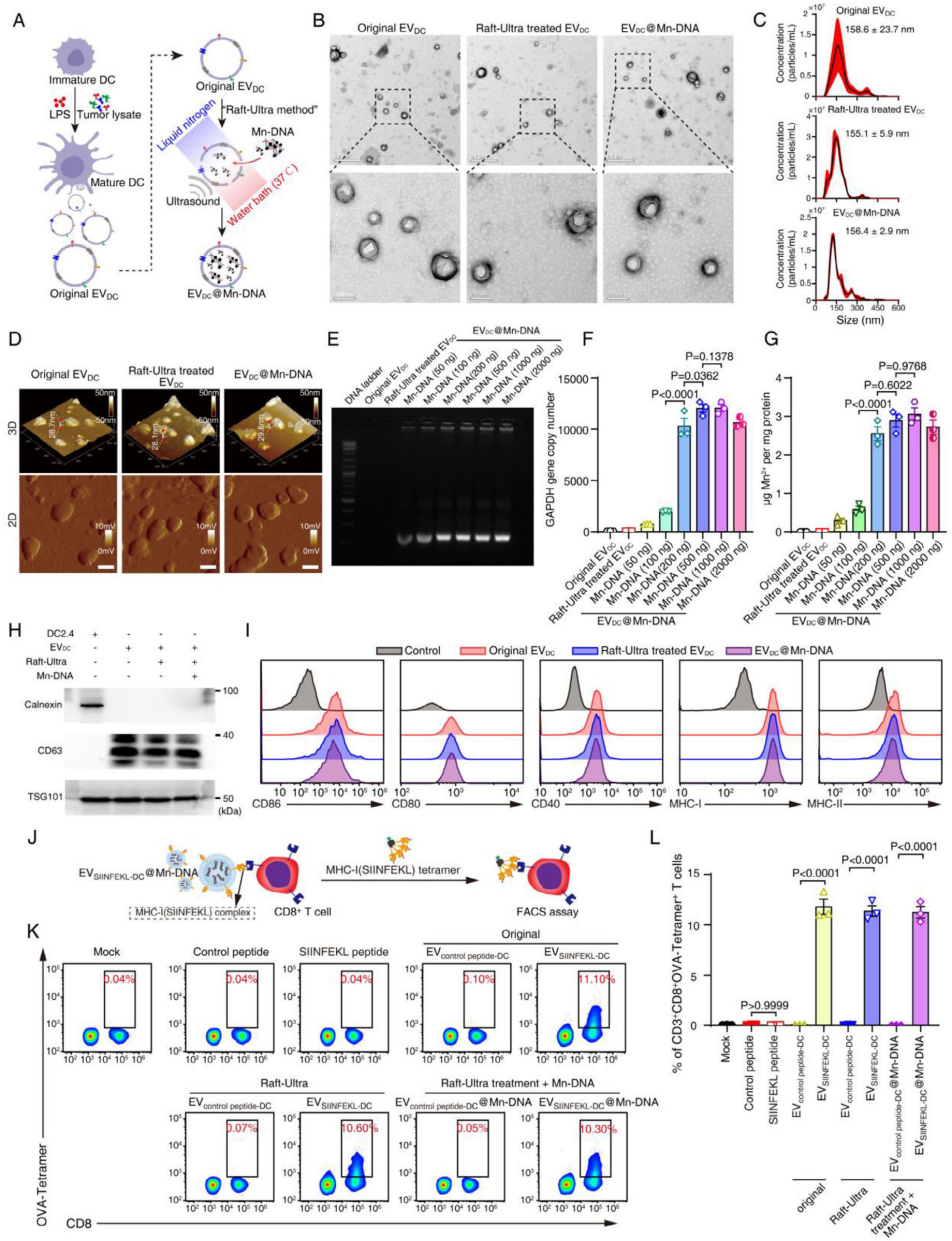
Generation and characterization of EV_DC_@Mn‐DNA. A) Schematic diagram illustrating the production of EV_DC_@Mn‐DNA using our newly developed Raft‐Ultra method. B) Transmission electron microscopy analysis of mouse dendritic cell derived EVs before and after receiving the Raft‐Ultra method preparation in the presence or absence of Mn‐DNA complexes. Magnified images are also given. C) Curve charts show the sizes of EV_DC_ before and after undergoing the Raft‐Ultra method preparation in the presence or absence of Mn‐DNA complexes. D) 2D and 3D AFM images of original EVs before and after Raft‐Ultra treatment, with or without Mn‐DNA, illustrating their morphology, thickness and structural features. Red arrows and dotted lines indicate EV thickness. E) Gel electrophoresis analysis of PCR product of GAPDH gene was performed in each group. F) Bar chart shows the GAPDH gene copy number in each group (n = 3 independent experiments). G) The bar chart shows the amount of Mn^2^⁺ (µg) per mg of protein in each group (n = 3 independent experiments). The highest Mn^2^⁺ loading per mg of protein was achieved when EV_DC_ were encapsulated with 200 ng of Mn‐DNA complexes. H) Western blot analysis of the indicated EV (CD63) and EV exclusion (calnexin) markers in each group. TSG101 was used as a loading control. I) FACS analysis of the expression of DC related maturation/identity markers CD86, CD80, CD40, MHC‐1 or MHC‐II on the surface of original EV_DC_, Raft‐Ultra treated EV_DC_ or EV_DC_@Mn‐DNA as compared to negative control. J) Diagram illustrates the working principle of OVA epitope SIINFEKL peptide pulsed DC‐derived EV encapsulating Mn‐DNA complex in priming CD8^+^T cells. K) FACS analysis was performed to assess the expression of the OVA epitope SIINFEKL‐specific TCR (T cell receptor) on CD3^+^CD8^+^ T cells following treatments. L) Bar chart displays the percentage of CD3^+^CD8^+^OVA‐Tetramer^+^ T cells relative to the total CD3^+^CD8^+^ T cell population in each group (n = 3 independent experiments). Results represent mean ± S.E.M. F, G, L) One‐way ANOVA with Tukey's post hoc test. Scale bars in (B) represent 0.5 µm (upper), 100 nm (lower). D) 100 nm.

Although Mn‐DNA complexes exhibit strong affinity for cGAS and effectively induce its phase separation, their potential for broader application is hindered by challenges such as non‐specific biodistribution, short half‐life, and the inability to traverse cell membranes, issues common in DNA‐based therapies.^[^ ^33^, ^34^ ^]^ Recent research has highlighted the potential of DC‐derived immunogenic extracellular vesicles (EVs) (including microvesicles) for delivering cellular contents to intratumoral DCs in mouse models.^[^ ^20^ ^]^ However, incorporating specific Mn‐DNA content into these EVs remains a technical challenge. To achieve encapsulation of Mn‐DNA complexes within tumor cell lysate–pulsed DC–secreted immunogenic EVs (EV_DC_), we developed a rapid freeze‐thaw–sonication method (Raft‐Ultra) in which EV_DC_ were mixed with Mn‐DNA, rapidly frozen in liquid nitrogen, quickly thawed at 37 °C, and subjected to sonication for three consecutive cycles (**Figure** **2**A). We next characterized the morphology of EV_DC_@Mn‐DNA using transmission electron microscopy (TEM) and nanoparticle tracking analysis (NTA), which showed membrane bound structures with no obvious difference in shape or particle size as compared to Raft‐Ultra method treated/untreated DC‐derived EVs (Figure 2B,C). 2D and 3D atomic force microscopy also revealed that the structural integrity of EV_DC_ was preserved before and after Raft‐Ultra treatment, with or without Mn‐DNA complexes, and that EV_DC_ thickness remained unchanged (Figure 2D). Quantification of GAPDH gene copies and Mn^2+^ levels revealed that the encapsulation efficiency of Mn‐DNA in EV_DC_ was higher at an input dose of 200 ng, reaching the optimal level (Figure 2E–G). Western blot analysis also indicated that there was no significant change in the expression of EV markers in EV_DC_ before and after the Raft‐Ultra treatment and packaging of Mn‐DNA complexes, and they exhibited reduced expression levels of the EV exclusion marker calnexin as compared to DC2.4 cells (Figure 2H). Additionally, FACS analysis showed no differences in the expression levels of co‐stimulatory molecules (CD86, CD80, CD40) and antigen presentation molecules (MHC‐I and MHC‐II) among original EV_DC_, Raft‐Ultra‐treated EV_DC_, and EV_DC_@Mn‐DNA (Figure 2I). This suggests that the packaging process of Mn‐DNA complexes did not compromise the membrane integrity of EV_DC_. Further FACS analysis showed that EV_DC_ with Mn‐DNA/DNA encapsulation or addition induced CD3⁺CD8⁺ T cell proliferation and activation at levels comparable to EV_DC_ alone, CD3/CD28 antibodies, or concanavalin A (Con. A) treatment (Figure S2A–D, Supporting Information), indicating that Raft‐Ultra treatment did not impair EV_DC_‐mediated regulation of CD8⁺ T cell proliferation and activity. To further demonstrate that the Raft‐Ultra treatment did not affect the antigen presentation ability of EV_DC_, we initially pulsed mouse immature DCs with either the OVA (ovalbumin) epitope SIINFEKL peptide (a well‐characterized immunogenic model epitope^[^ ^35^ ^]^) or a control peptide, and then stimulated them to secrete EV_SIINFEKL‐DC_ or EV_control peptide‐DC_, which were subsequently used to encapsulate Mn‐DNA complexes (Figure 2J). FACS analysis indicated that treatment with EV_SIINFEKL‐DC_, Raft‐Ultra treated EV_SIINFEKL‐DC_, or EV_SIINFEKL‐DC_@Mn‐DNA all resulted in increased expression of the OVA epitope SIINFEKL‐specific TCR (T‐cell receptor) in CD3^+^CD8^+^T cells compared to the other control groups, with no noticeable difference in the upregulation of the OVA epitope SIINFEKL‐specific TCR between them (Figure 2K,L), confirming that the T cell priming ability of EV_DC_ was not affected by the Raft‐Ultra process. Overall, these findings demonstrate that EV_DC_ could effectively encapsulate Mn‐DNA complexes through the Raft‐Ultra method and preserved the antigen presentation ability of activated DCs to prime T cells.

### 2.3 EV_DC_@Mn‐DNA Delivers Mn‐DNA Complexes to the Cytoplasm through Membrane Fusion, Triggering cGAS Phase Condensation

**Figure 3:**
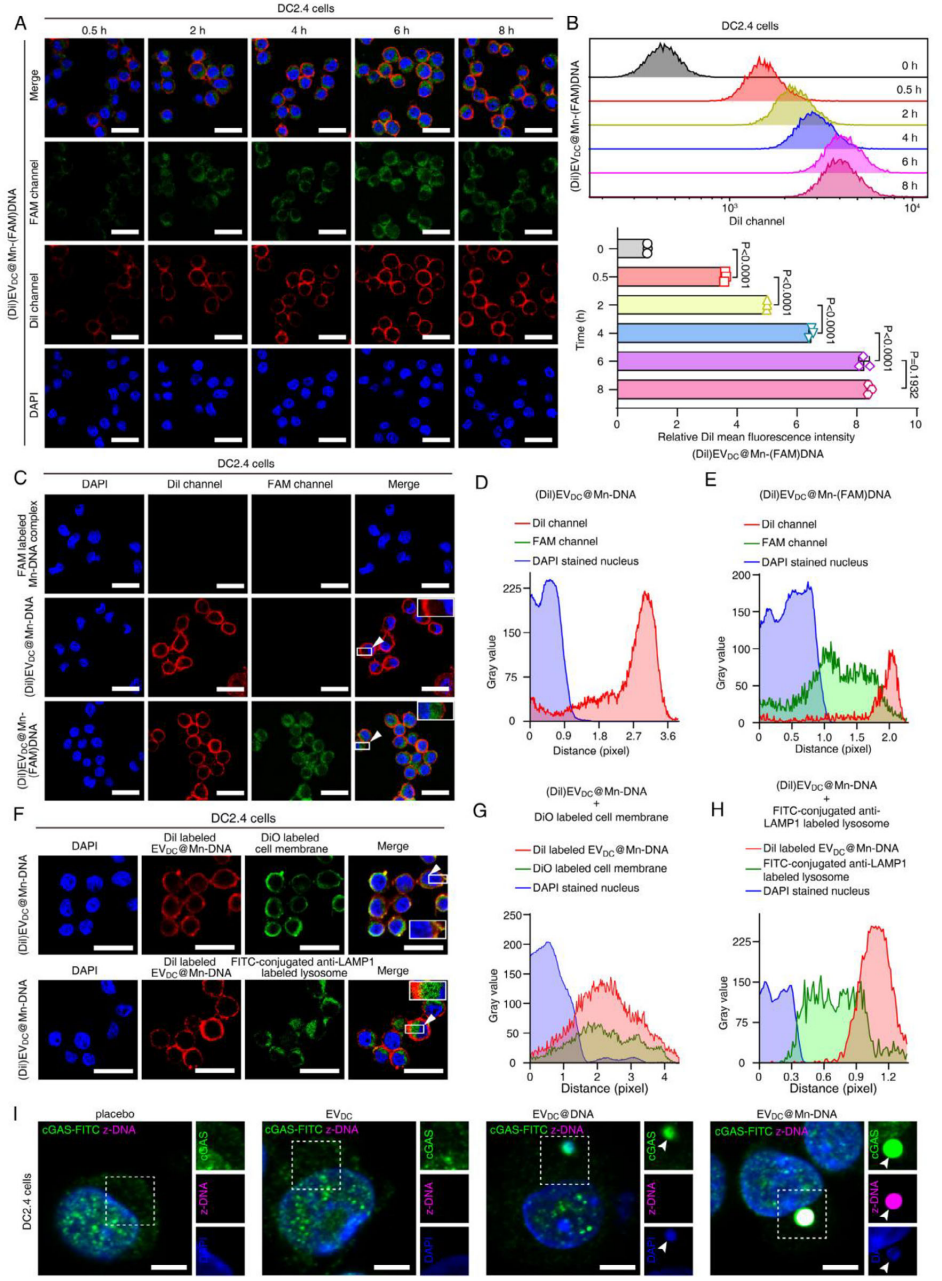
EV_DC_@Mn‐DNA is uptaken by dendritic cells through membrane fusion. A) Representative images of mouse DCs after treated with Dil (red) labeled EV_DC_ encapsulating Mn‐FAM (green) labeled DNA over time. DAPI stained nuclei. B) FACS analysis of mouse DCs after treated with (Dil)EV_DC_@Mn‐(FAM)DNA over time. Bar chart shows the relative mean Dil fluorescence intensity in each group over time (n = 3 independent experiments). C) Representative images of mouse DCs after treated with FAM labeled Mn‐DNA complex, (Dil)EV_DC_@Mn‐DNA or (Dil)EV_DC_@Mn‐(FAM)DNA for 6 h are given (n = 3 independent experiments). White boxes indicate the magnified areas. D,E) Distribution graph illustrates the distance pixel between Dil channel (red), FAM channel (green) and DAPI‐stained nucleus (blue) in mouse DCs following treatment with (Dil)EV_DC_@Mn‐DNA or (Dil)EV_DC_@Mn‐(FAM)DNA. Gray value represents the intensity of each fluorescence signal. F) Representative confocal images of DiO or FITC conjugated anti‐LAMP1 stained DCs after treated with (Dil)EV_DC_@Mn‐DNA are given (n = 3 independent experiments). White boxes indicate the magnified areas. G,H) Distribution graph illustrates the distance pixel between Dil channel (red), DiO channel/FITC conjugated anti‐LAMP1 stained lysosome (green) and DAPI‐stained nucleus (blue) in (Dil)EV_DC_@Mn‐DNA treated mouse DCs. Gray value represents the intensity of each fluorescence signal. I) Representative fluorescent images of cGAS, Z‐DNA, and DAPI staining in DCs following the indicated treatments are provided. Magnified views are displayed on the right‐hand side, with white arrows highlighting cGAS phase separation. Results represent mean ± S.E.M. B) One‐way ANOVA with Tukey's post hoc test. Scale bars in (A, C, F) represent 50 µm. I) 5 µm.

To examine the cellular uptake behavior of EV_DC_@Mn‐DNA by mouse dendritic cell line DC2.4, we prepared fluorescently labeled EV_DC_@Mn‐DNA in which DNA was first labeled with green fluorescent dye FAM (fluorescein‐labeled oligonucleotide probes) and then coordinated with Mn^2+^, and the phospholipid bilayer of EV_DC_ was stained with the red fluorescent lipophilic membrane dye Dil, followed by confocal imaging and FACS analysis. The results showed that EV_DC_@Mn‐DNA could be rapidly taken up by DC2.4 cells over time and reached the peak after 6 h (**Figure** **3**A,B). During this period, we observed that the red fluorescence signal of the membrane did not co‐localize with the green fluorescence signal of DNA after EV_DC_@Mn‐DNA was taken up by DCs (Figure 3A). To exclude interference from free Mn‐DNA complexes on cellular uptake behavior, we separately co‐incubated DCs for 6 h with FAM labeled Mn‐DNA complex, Dil single‐color labeled EV_DC_@Mn‐DNA ((Dil)EV_DC_@Mn‐DNA), and Dil labeled EV_DC_ encapsulating FAM labeled Mn‐DNA complex ((Dil)EV_DC_@Mn‐(FAM)DNA), and subjected to immunofluorescent imaging and FACS analysis (Figure 3C–E). The results showed that the FAM green fluorescently labeled free Mn‐DNA complex could not be taken up by DCs directly, whereas no green FAM fluorescent signal was observed after Dil single‐color labeled EV_DC_@Mn‐DNA was taken up by DCs (Figure 3C–E). In contrast, the green signal was only observed in DCs after treated with (Dil)EV_DC_@(FAM)Mn‐DNA, with almost no overlapping between the red and green fluorescent signals (Figure 3C–E). The results confirmed the effective delivery of Mn‐DNA into the cytoplasm of DCs using EV_DC_ as a carrier. To investigate the pathways through which DCs uptake EV_DC_@Mn‐DNA, mouse DCs were labeled with DiO for the cell membrane and FITC‐conjugated anti‐LAMP1 antibody for lysosomes. Upon uptake by DC2.4 cells, the Dil‐red signal of EV_DC_@Mn‐DNA did not co‐localize with lysosomes but was observed at the cell membrane (Figure 3F–H). To further validate this finding, mouse DC2.4 cells treated with EV_DC_@(FAM)Mn‐DNA were immunostained with anti‐LAMP1, and no co‐localization between lysosomes and FAM‐labeled Mn‐DNA was detected (Figure S3, Supporting Information). To further evaluate the capability of EV_DC_ to deliver DNA or Mn‐DNA complexes into the cytoplasm, we incubated DCs with EV_DC_@DNA and EV_DC_@Mn‐DNA, followed by staining for cGAS, Z‐DNA and DAPI. The results showed that cGAS phase‐separated droplets co‐localized with DAPI fluorescence were detected in the EV_DC_@DNA group, but no Z‐DNA signal was observed. In contrast, the EV_DC_@Mn‐DNA group exhibited cGAS phase‐separated droplets co‐localized with both DAPI and Z‐DNA, indicating that EV_DC_@Mn‐DNA can deliver Mn‐DNA into cells and form phase‐separated droplets with cGAS without altering the Z‐DNA conformation of Mn‐DNA complexes (Figure 3I). Notably, the cGAS phase‐separated droplets were larger in DCs treated with EV_DC_@Mn‐DNA compared to those treated with EV_DC_@DNA (Figure 3I). Overall, these results demonstrate that EV_DC_@Mn‐DNA efficiently delivers Mn‐DNA complexes into the cytoplasm probably through membrane fusion with DCs, preventing lysosomal degradation. This enhances cGAS recognition of Mn‐DNA complexes and promotes its phase condensation.

### 2.4 EV_DC_@Mn‐DNA Treatment Enhances cGAS‐STING‐Mediated DC Maturation and Tumor Antigen Presentation

**Figure 4:**
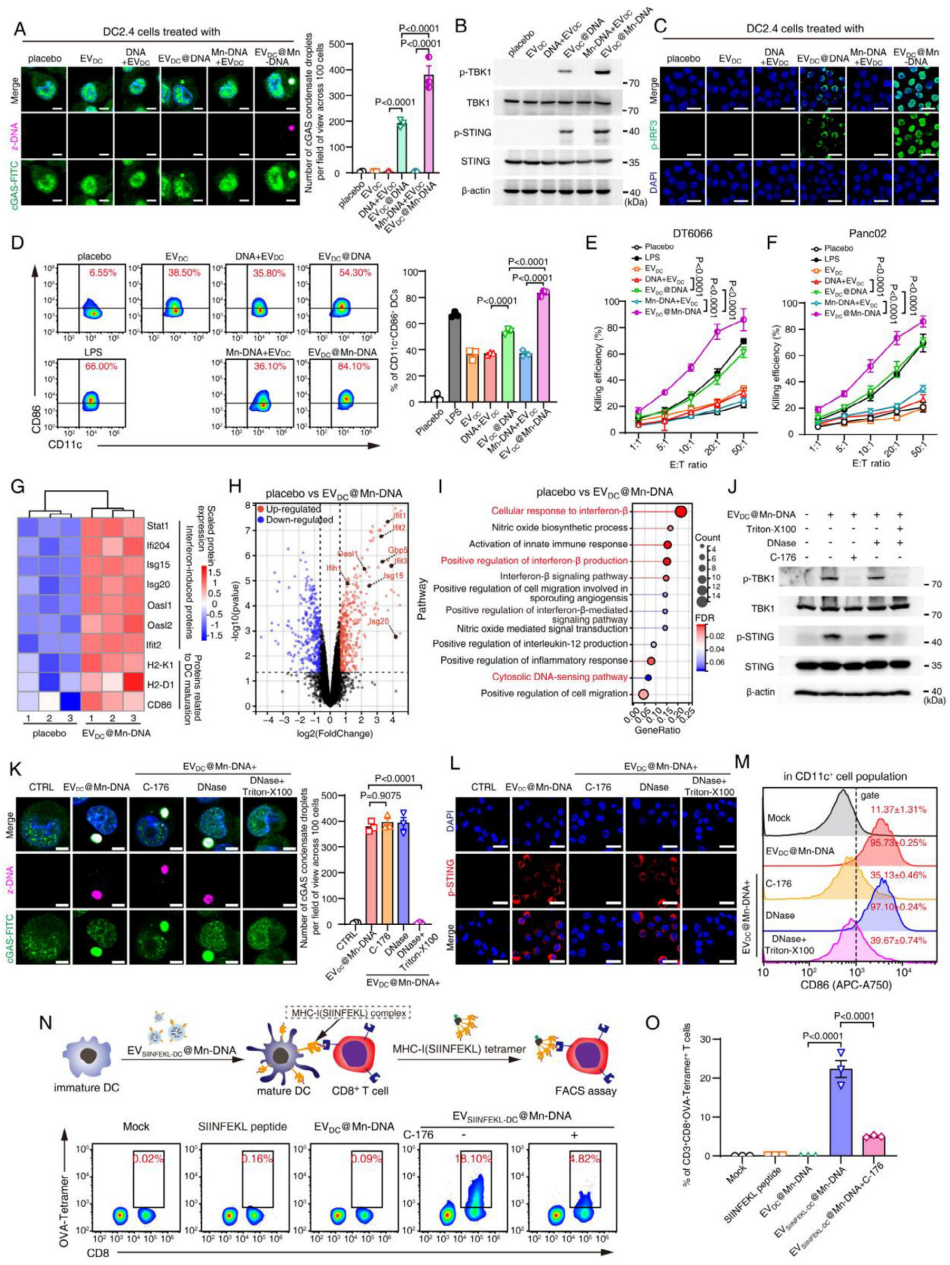
EV_DC_@Mn‐DNA treatment effectively activates the cGAS‐STING pathway mediated DC maturation. A) Representative images of cGAS, Z‐DNA, and DAPI staining in DCs after treatment are shown. White arrows indicate cGAS phase separation. Bar chart shows the number of cGAS condensate droplets per field of view across 100 DC2.4 cells. (n = 3 independent experiments). B) Western blot analysis of the cGAS‐STING pathway related proteins in mouse DCs following the indicated treatments. β‐actin was used as loading control (n = 3 independent experiments). C) Representative images of p‐IRF3 immunostaining on mouse DCs in each group (n = 3 independent experiments). D) FACS analysis of DC maturation marker CD86 expression in mouse CD11c^+^DCs after treated with the indicated treatments. Bar chart shows the percentage of CD11c^+^CD86^+^ DCs in the total CD11c^+^ DC population in each group (n = 3 independent experiments). E,F) Cytotoxicity LDH release assays on DT6066 or Panc02 cells were conducted using different ratios of CD3^+^CD8^+^ T cells that had been co‐cultured with DCs pre‐treated with the indicated treatments (n = 3 independent experiments). G) Heatmap analysis of the proteomics data of placebo or EV_DC_@Mn‐DNA treated DC2.4 cells (n = 3 independent samples). H) Volcano plot analysis of the proteomics data in G. The up‐regulated proteins involved in the cGAS‐STING pathway were marked in red. I) KEGG enrichment pathway analysis of the proteomics data in G. The pathways related to cGAS‐STING pathway were highlighted in red. (J) Western blot analysis of the indicated proteins in each group (n = 3 independent experiments). K) Representative images of cGAS, Z‐DNA, and DAPI staining in DCs after treatment are shown. Bar chart shows the number of cGAS condensate droplets per field of view across 100 cells. L) Representative confocal images of p‐STING immunostaining on DCs in each group are given. M) FACS analysis of CD86 expression in DCs following the indicated treatments. Red words indicate the mean percentage of CD11c^+^CD86^+^ DCs in the total CD11c^+^ DC population in each group. N) Schematic diagram showing the operational concept of EV_SIINFEKL‐DC_@Mn‐DNA pulsed DCs for priming CD8^+^ T cells and subsequent analysis via flow cytometry. FACS analysis of the expression of OVA‐tetramer in CD3^+^CD8^+^ T cells after treated with the indicated treatments. O) Bar chart shows the percentage of CD3^+^CD8^+^OVA‐tetramer^+^ T cells in the total CD3^+^CD8^+^ T cell population in each group (n = 3 independent experiments). Results represent mean ± S.E.M. A, D, K, O) One‐way ANOVA with Tukey's post hoc test. E, F) Two‐way ANOVA with Tukey's post hoc test. Scale bars in (A, K) represent 5 µm. C, L) 50 µm.

To investigate the role of Mn‐DNA in DC activation delivered by EV_DC_@Mn‐DNA, we included control groups in which Mn‐DNA complexes or DNA alone were simply mixed with EV_DC_ prior to co‐culture with DC2.4 cells. Cells were treated with placebo, EV_DC_, DNA plus EV_DC_ (DNA alone), EV_DC_@DNA (EV_DC_ encapsulating DNA alone), Mn‐DNA plus EV_DC_, or EV_DC_@Mn‐DNA for 6 h, followed by confocal imaging and western blot analysis. The mixture groups (DNA or Mn‐DNA plus EV_DC_) were included to demonstrate that encapsulation via the Raft‐Ultra method, rather than simple mixing, is critical for intracellular delivery. Notably, phase‐separated cGAS droplets were most prominently observed in DCs treated with EV_DC_@Mn‐DNA, even compared with EV_DC_@DNA, whereas no phase separation occurred in the other groups, including DNA plus EV_DC_ or Mn‐DNA plus EV_DC_. These results indicate that EV_DC_@Mn‐DNA delivers Mn‐DNA intracellularly through encapsulation within EVs rather than via surface adsorption in the mixture groups, highlighting the essential role of EV‐mediated delivery for cGAS activation and DC maturation (**Figure** **4**A). Western blot analysis showed that EV_DC_@Mn‐DNA treatment markedly increased phosphorylation of STING and TBK1 in mouse DC2.4 cells compared with EV_DC_@DNA, whereas EV_DC_ alone or Mn‐DNA/DNA plus EV_DC_ had no significant effect on the cGAS‐STING pathway (Figure 4B). Further immunostaining revealed that EV_DC_@Mn‐DNA treatment induced the most pronounced accumulation of phosphorylated IRF3 (p‐IRF3), a downstream effector of TBK1, in the nuclei of DCs compared with all other treatment groups, including EV_DC_@DNA (Figure 4C).

To explore the relationship between cGAS‐STING activation and DC maturation, mouse bone marrow‐derived dendritic cells were then treated with placebo, lipopolysaccharide (LPS), EV_DC_, DNA plus EV_DC_, EV_DC_@DNA, Mn‐DNA plus EV_DC_, or EV_DC_@Mn‐DNA. FACS analysis showed that EV_DC_@Mn‐DNA treatment induced the largest increase in the proportion of CD11c⁺CD86⁺ DCs, indicating enhanced DC maturation compared with all other treatment groups, including EV_DC_@DNA and the LPS positive control group (Figure 4D). Furthermore, naïve CD8⁺ T cells pulsed with these pre‐treated DCs exhibited enhanced cytotoxicity against the DT6066 cells, with T cells primed by EV_DC_@Mn‐DNA–treated DCs demonstrating higher killing efficiency than those pulsed with EV_DC_@DNA or lipopolysaccharide (LPS)‐treated DCs (Figure 4E). This effect was further validated using another mouse pancreatic cancer cell line, Panc02, yielding consistent results (Figure 4F). To further confirm our observations in a human model and increase the clinical significance of our findings, we generated human Panc1 cancer cell lysate–pulsed human DC (hDC)–derived EVs (termed as EV_hDC_) and encapsulated them with manganese–human Panc1 pancreatic cancer cell–derived DNA complexes (EV_hDC_@Mn‐DNA) using the method described above. These EVs were then used to treat human DCs and analyzed by FACS. The results showed that EV_hDC_@Mn‐DNA treatment markedly enhanced human DC maturation, compared with other groups, including EV_hDC_@DNA alone (Figure S4A,B, Supporting Information). To investigate the molecular mechanism of EV_DC_@Mn‐DNA mediated DC maturation, proteomics analysis of DC2.4 cells was performed after treatment with either placebo or EV_DC_@Mn‐DNA. Both heatmap and volcano plot analysis of the proteomics data demonstrated up‐regulation of interferon‐induced proteins including Stat1, Ifi204, Isg15, Isg20, Oasl1, Oasl2 and lfit2 as well as costimulatory molecules such as CD86 and H2‐K1 and H2‐D1 in DC2.4 cells treated with EV_DC_@Mn‐DNA compared to placebo treated cells (Figure 4G,H). Kyoto Encyclopedia of Genes and Genomes (KEGG) enrichment analysis of the proteomics data revealed the enrichment of pathways such as cellular response to interferon‐β, positive regulation of interferon‐β production, cytosolic DNA‐sensing pathway, and others in EV_DC_@Mn‐DNA treated DC2.4 cells compared to the placebo treated group (Figure 4I). To determine the significance of either surface‐adsorbed DNA or entrapped DNA in EV_DC_@Mn‐DNA in regulating the cGAS‐STING signaling pathway in DCs, digestion of surface or internal DNA in EV_DC_@Mn‐DNA was performed using DNase or DNase plus the membrane‐disrupting agent Triton X‐100. DCs were also treated with or without EV_DC_@Mn‐DNA in the presence or absence of the STING inhibitor C‐176 to assess whether activation occurred via the cGAS‐STING pathway. The DCs were then analyzed using western blotting, immunofluorescent imaging, and flow cytometry. The findings revealed that EV_DC_@Mn‐DNA lost its ability to induce cGAS phase separation when pre‐treated with Triton X‐100 for membrane disruption and DNase for internal DNA degradation. Additionally, it failed to activate the cGAS‐STING pathway and induce DC maturation when subjected to the same pre‐treatment or in the presence of a STING inhibitor. In contrast, DNase treatment alone had no effect on the cGAS phase separation or the immunostimulatory functions of EV_DC_@Mn‐DNA (Figure 4J–M). We then investigated whether EV_DC_@Mn‐DNA was capable to transfer their MHC‐tumor antigen complex to immature DCs. DC2.4 cells were initially pulsed with the OVA epitope SIINFEKL peptide to generate EV_SIINFEKL‐DC_, which were subsequently used to encapsulate Mn‐DNA complexes. These EV_SIINFEKL‐DC_@Mn‐DNA were then utilized to pulse mouse immature DCs, followed by co‐culture with CD3^+^CD8^+^ T cells in the presence or absence of the STING inhibitor C‐176 (Figure 4N,O). FACS analysis revealed that treatment with EV_SIINFEKL‐DC_@Mn‐DNA successfully transferred the MHC‐OVA epitope complex to DCs, likely through membrane fusion. This was evidenced by the increased expression of the OVA epitope SIINFEKL‐specific TCR in CD3^+^CD8^+^ T cells after co‐culture with EV_SIINFEKL‐DC_@Mn‐DNA pulsed DCs as compared to other groups. In contrast, STING inhibitor reduced the expression of the OVA epitope SIINFEKL‐specific TCR in CD3^+^CD8^+^ T cells co‐culture with EV_SIINFEKL‐DC_@Mn‐DNA pretreated DCs (Figure 4N,O). Overall, these findings suggest that EV_DC_@Mn‐DNA depends on internally contained Mn‐DNA complexes to activate the cGAS‐STING pathway in DCs, thereby promoting their maturation and antigen presentation ability.

### 2.5 EV_DC_@Mn‐DNA Treatment Activates Anti‐Tumor Immune Response Against Orthotopic Pancreatic Tumor Growth

**Figure 5:**
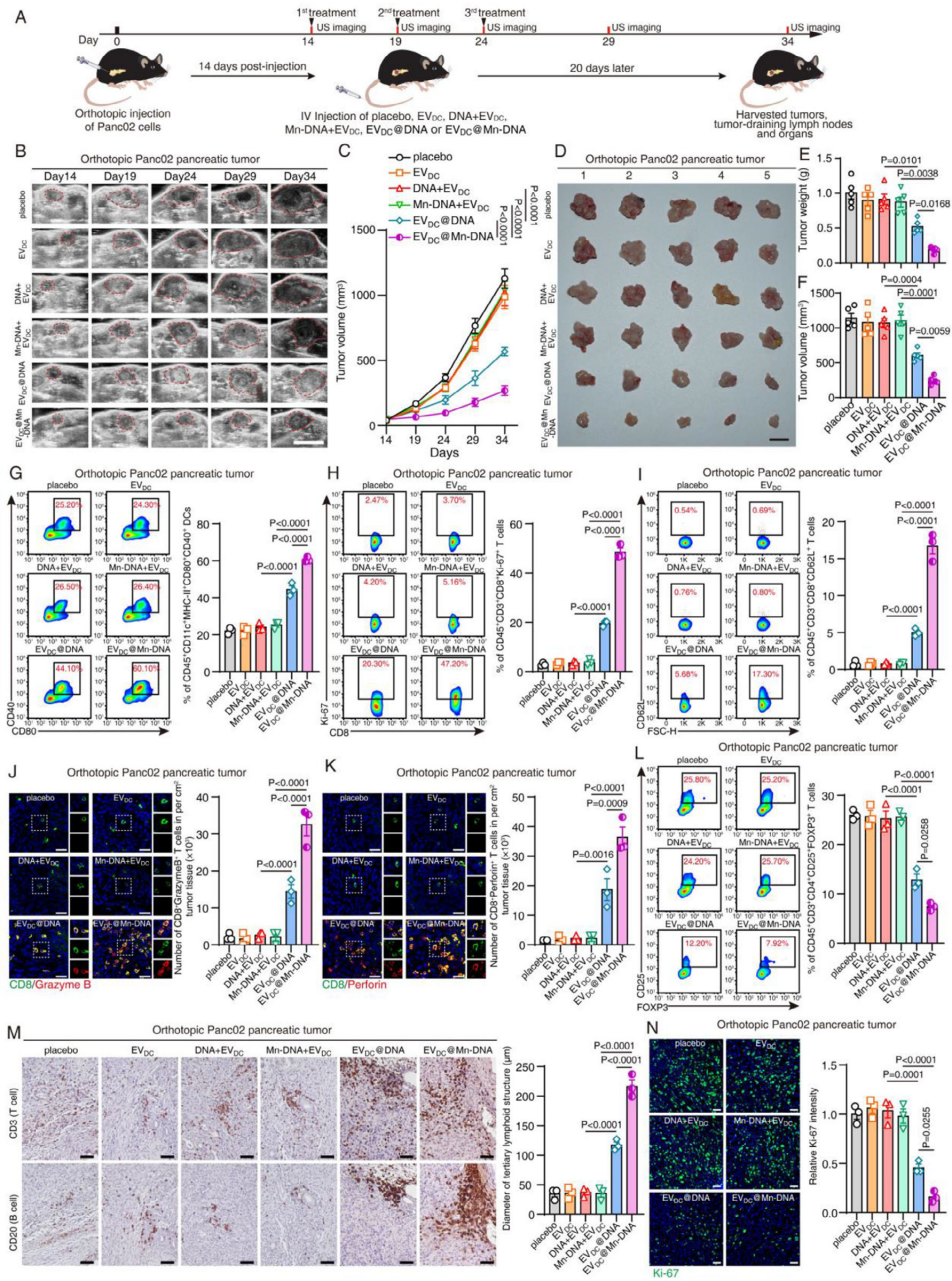
EV_DC_@Mn‐DNA treatment induces anti‐tumor immune response against orthotopic Panc02 pancreatic tumor growth. A) Schematic diagram showing the treatment and ultrasound (US) imaging schedule of orthotopic Panc02 pancreatic tumor bearing mice. B) Representative ultrasound images of orthotopic Panc02 pancreatic tumor growth over time in each group are given. Red dotted line indicates tumor position. C) Line graph shows the growth curve of orthotopic Panc02 pancreatic tumors in each group (n = 5 mice per group). D) A tumor gross image from each group is provided. E,F) Bar chart shows the final tumor weight or volume in each group (n = 5 mice per group). G) FACS analysis of the expression of CD40/CD80 in CD45^+^CD11c^+^MHC‐II^+^DCs from orthotopic Panc02 pancreatic tumors following the indicated treatments. Bar chart shows the percentage of CD45^+^CD11c^+^MHC‐II^+^CD80^+^CD40^+^ DCs in the total CD45^+^CD11c^+^MHC‐II^+^ DC population in each group (n = 3 mice per group). H,I) FACS analysis of the expression of proliferation marker Ki‐67 or central memory marker CD62L in CD45^+^CD3^+^CD8^+^ T cells from orthotopic Panc02 pancreatic tumors following the indicated treatments. Bar chart shows the percentage of CD45^+^CD3^+^CD8^+^Ki‐67^+^ or CD45^+^CD3^+^CD8^+^CD62L^+^ in the total CD45^+^CD3^+^CD8^+^ T cell population in each group (n = 3 mice per group). J,K) Representative images of granzyme B/perforin and CD8 co‐immunostaining on tumor sections derived from each group are given. L) FACS analysis of regulatory T cell markers CD25 and FOXP3 in CD45^+^CD3^+^CD4^+^ T cells from orthotopic pancreatic tumors following the indicated treatments. Bar chart shows the percentage of CD45^+^CD3^+^CD4^+^CD25^+^FOXP3^+^ T cells in the total CD45^+^CD3^+^CD4^+^ T cell population in each group (n = 3 mice per group). M) Representative immunohistochemical stained images of CD3/CD20 on serial tumor sections derived from each group are given. Bar chart shows the diameter of tertiary lymphoid structure in each group (n = 3 mice per group). N) Representative images of Ki‐67 immunostaining on tumor sections derived from each group are given. Bar chart shows the relative Ki‐67 intensity in each group (n = 3 mice per group). Results represent mean ± S.E.M. C) Two‐way ANOVA with Tukey's post hoc test. E–N) One‐way ANOVA with Tukey's post hoc test. Scale bars in (B, D) represent 1 cm. J, K, M, N) 50 µm.

To assess the antitumor efficacy of EV_DC_@Mn‐DNA in pancreatic cancer, we established an orthotopic pancreatic cancer model by injecting Panc02 cells into the pancreas of C57BL/6 mice. Tumor‐bearing mice were then treated with placebo, EV_DC_, DNA plus EV_DC_, EV_DC_@DNA, Mn‐DNA plus EV_DC_, or EV_DC_@Mn‐DNA, and tumor growth was monitored using a 3D ultrasound imaging system (**Figure** **5**A). EV_DC_@Mn‐DNA most effectively suppressed tumor growth compared to all other groups, including EV_DC_@DNA, whereas Mn‐DNA/DNA plus EV_DC_ or EV_DC_ alone showed no significant effect relative to placebo (Figure 5B–F). To evaluate immune responses following treatment, we performed immunofluorescence (IF) staining on tumor sections and analyzed digested tumors and tumor‐draining lymph nodes by FACS 10 days after the last treatment, at the study endpoint. The gating strategy for immune cell subsets is shown in Figure S5A, Supporting Information. EV_DC_@Mn‐DNA markedly increased total intratumoral DCs and their activation/maturation, with a higher proportion of activated DCs within the cDC1 (conventional type 1 DC) subset than in cDC2 in both tumors and tumor‐draining lymph nodes, compared to other treatments, including EV_DC_@DNA. (Figure 5G and Figure S5B–E, Supporting Information). EV_DC_@Mn‐DNA also markedly increased CD3⁺CD8⁺ T cells, including Ki‐67⁺ proliferating T cells (with Ki‐67 serving as a marker of proliferation) and CD62L⁺ central memory T cells, in Panc02 tumors compared to all other treatment groups (Figure 5H,I and Figure S5F, Supporting Information). Immunofluorescence confirmed an increased population of CD8⁺Granzyme B⁺/Perforin⁺ T cells (Figure 5J,K). Consistent with reports that activated DCs secrete CXCL9 and CCL5 to recruit DCs and T cells,^[^ ^36^ ^]^ EV_DC_@Mn‐DNA treatment elevated CXCL9⁺ and CCL5⁺ DCs in tumors (Figure S5G,H, Supporting Information). Given the immunosuppressive role of regulatory T cells (Tregs) in pancreatic cancer,^[^ ^37^ ^]^ we quantified intratumoral Tregs and found that EV_DC_@Mn‐DNA more effectively reduced their population compared to EV_DC_@DNA (Figure 5L). Interestingly, tertiary lymphoid structure presence or size may correlate with antitumor immune responses in pancreatic cancer.^[^ ^38^ ^]^ Immunohistochemistry showed that EV_DC_@Mn‐DNA treatment markedly increased the size of tertiary lymphoid structures in tumors as compared to the EV_DC_@DNA group, whereas Mn‐DNA/DNA plus EV_DC_ or EV_DC_ alone had no effect relative to placebo (Figure 5M). Supporting its antitumor activity, immunostaining showed a greater reduction in Ki‐67⁺ tumor cells following EV_DC_@Mn‐DNA treatment compared to other groups (Figure 5N).

To further validate our observations, we employed a second orthotopic pancreatic tumor model by implanting DT6066 cells into C57BL/6 mice and monitored tumor growth under different treatments using ultrasound imaging (Figure S6A, Supporting Information). EV_DC_@Mn‐DNA treatment most effectively suppressed tumor growth compared with all other groups, including EV_DC_@DNA (Figure S6B–F, Supporting Information). FACS and IF analyses showed that EV_DC_@Mn‐DNA treatment increased intratumoral DCs and enhanced their maturation, with a higher proportion of activated DCs in the cDC1 subset compared with cDC2, in both tumors and tumor‐draining lymph nodes, relative to other treatments, including EV_DC_@DNA. (Figure S6G–K, Supporting Information). This was accompanied by increased CD8⁺ T cell infiltration, proliferation, central memory characteristics, and activation (Figure S7A–E, Supporting Information). Moreover, EV_DC_@Mn‐DNA promoted higher expression of CXCL9 and CCL5 in DCs (Figure S7F,G), reduced intratumoral Tregs (Figure S7H, Supporting Information), and enlarged tertiary lymphoid structures in DT6066 pancreatic tumors (Figure S7I, Supporting Information). Finally, Ki‐67 staining showed reduced tumor cell proliferation following EV_DC_@Mn‐DNA treatment (Figure S7J, Supporting Information). To assess the biodistribution of EV_DC_@Mn‐DNA in tumor‐bearing mice, we performed in vivo and ex vivo near‐infrared fluorescence imaging following intravenous injection of DiR‐labeled EV_DC_@Mn‐DNA into DT6066 subcutaneous tumor‐bearing mice. The results showed predominant tumor accumulation over time compared with mice injected with DiR dye alone (Figure S8A, Supporting Information). Ex vivo imaging further confirmed greater tumor accumulation of EV_DC_@Mn‐DNA relative to the control (Figure S8B, Supporting Information). Notably, hematoxylin‐eosin (H&E) staining of major organs indicated that EV_DC_@Mn‐DNA treatment had no obvious organ‐specific toxicity in orthotopic Panc02 and DT6066 pancreatic tumor‐bearing mice as compared to placebo treated group (Figure S9A,B, Supporting Information), while it also did not affect average mouse body weight during the treatment period (Figure S9C,D, Supporting Information). Overall, these findings suggest that our EV_DC_@Mn‐DNA treatment can elicit a robust anti‐tumor immune response, even in the context of pancreatic tumors that are traditionally resistant to immunotherapy.

### 2.6 EV_DC_@Mn‐DNA Treatment Restores Vascular‐Immune Homeostasis in Orthotopic Pancreatic Tumors

**Figure 6:**
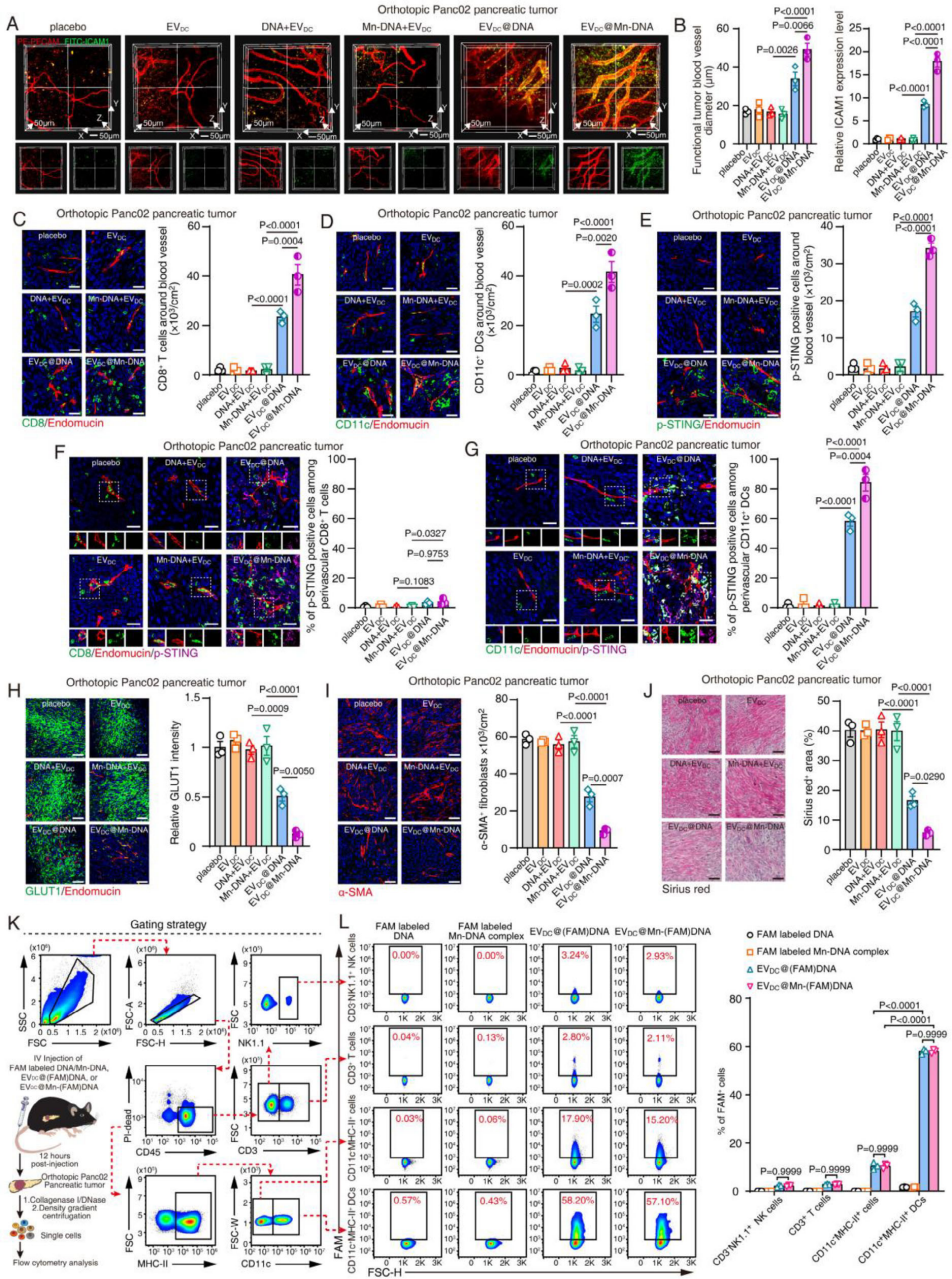
EV_DC_@Mn‐DNA uptake by endogenous DCs restores vascular‐immune homeostasis, favoring immune cell extravasation. A) Representative 3D images of in vivo multi‐photon confocal imaging on orthotopic Panc02 pancreatic tumors from each treatment group are given. Split images are shown below. B) Bar chart shows the diameters of functional blood vessels (left) or relative ICAM1 expression level (right) in each group (n = 3 mice per group). C) Representative images of endomucin and CD8 co‐immunostaining on tumor sections derived from each group are given. Bar chart shows the number of CD8^+^ T cells ×10^3^ cm^−2^ around blood vessels in each group. D) Representative images of endomucin and CD11c co‐immunostaining on tumor sections derived from each group are given. Bar chart shows the number of CD11c^+^ DCs ×10^3^ cm^−2^ around blood vessels in each group. E) Representative images of endomucin and p‐STING co‐immunostaining on tumor sections derived from each group are given. Bar chart shows the number of p‐STING positive cells ×10^3^ cm^−2^ around blood vessels in each group. F,G) Representative images of endomucin, p‐STING and CD8/CD11c triple‐immunostaining on tumor sections derived from each group are given. Bar charts show the percentage of p‐STING positive cells among perivascular CD8^+^ T cells/CD11c^+^ DCs in each group. H) Representative images of endomucin and GLUT1 co‐immunostaining on tumor sections derived from each treatment group are given. Bar chart shows the relative GLUT1 intensity in each group (n = 3 mice per group). I) Representative images of α‐SMA immunostaining on tumor sections derived from each treatment group are given. Bar chart shows the number of α‐SMA positive fibroblasts ×10^3^ cm^−2^ in each group (n = 3 mice per group). J) Representative images of Sirius red staining on tumor sections in each group are given. Bar chart shows the percentage of Sirius red positive area in each group (n = 3 mice per group). K,L) FACS analysis of the expression of markers for NK cell, T cell, CD11c^−^MHC‐II^+^ cells and CD11c^+^MHC‐II^+^ DCs, as well as FAM intensity, in orthotopic Panc02 pancreatic tumors from each group, accompanied by a gating strategy and schematic diagram for the procedure. Bar chart shows the percentage of FAM‐stained positive cells in each cell type. Results represent mean ± S.E.M. B–J) One‐way ANOVA with Tukey's post hoc test. L) Two‐way ANOVA with Tukey's post hoc test. Scale bars in (A, C–J) represent 50 µm.

Recent studies have highlighted the significance of adhesion molecules like ICAM1 (intercellular adhesion molecule 1) in facilitating immune cell extravasation/transendothelial migration into tumor tissues, thus influencing solid tumor responses to immunotherapy.^[^ ^39^ ^]^ Additionally, there is emerging evidence suggesting that activated DCs might influence vasculature remodeling through paracrine signaling,^[^ ^13^ ^]^ although the exact mechanism remains unclear. To explore this, FITC‐conjugated ICAM1 antibody was intravenously injected into orthotopic Panc02 or DT6066 pancreatic tumor‐bearing mice, which had received three consecutive treatments of placebo, EV_DC_, DNA plus EV_DC_, EV_DC_@DNA, Mn‐DNA plus EV_DC_ or EV_DC_@Mn‐DNA, and PE‐PECAM antibody was used to label functional tumor blood vessels (Figure S10A, Supporting Information). Multi‐photon microscopy imaging showed that the diameter of functional tumor blood vessels and the expression level of ICAM1 in functional tumor blood vessels was increased in EV_DC_@Mn‐DNA treated mice as compared to placebo, EV_DC_ alone, Mn‐DNA/DNA plus EV_DC_ and even EV_DC_@DNA (**Figures** **6**A,B and S10B, C, Supporting Information). Additionally, IF experiments showed an elevated number of CD8^+^ T cells and CD11^+^ DCs around the blood vessels in tumors derived from the mice that were treated with EV_DC_@Mn‐DNA as compared to other treatment groups, including EV_DC_@DNA group (Figure 6C,D and Figure S10D, E, Supporting Information). To validate the enhanced vascular function in orthotopic Panc02 and DT6066 tumors, we performed microbubble contrast‐enhanced ultrasound imaging in orthotopic Panc02 or DT6066 tumor bearing mice subjected to the treatments described above. EV_DC_@Mn‐DNA markedly increased tumor blood vessel flow and perfusion compared to other groups, including EV_DC_@DNA (Figure S11A–D, Supporting Information). To assess the effect on tumor vascular drug delivery, fluorescein‐labeled PD‐L1 antibody was administered intravenously to orthotopic Panc02 or DT6066 tumor–bearing mice treated with either placebo or EV_DC_@Mn‐DNA. Mice were subsequently injected with PE–PECAM antibody, and multi‐photon imaging was performed. EV_DC_@Mn‐DNA treatment enhanced intratumoral delivery of fluorescein‐labeled PD‐L1 antibody over time compared with the placebo group in both tumor models (Figure S11E–G, Supporting Information). To assess the impact of EV_DC_@Mn‐DNA treatment on CD8^+^ T cell recruitment, we intravenously injected CFSE green fluorescently labeled CD8^+^ T cells into orthotopic Panc02 or DT6066 tumor bearing mice treated with placebo, EV_DC_, DNA plus EV_DC_, EV_DC_@DNA, Mn‐DNA plus EV_DC_ or EV_DC_@Mn‐DNA. Subsequently, we administered PE‐PECAM antibody intravenously and conducted multi‐photon microscopy imaging. The results revealed an enhanced intratumor infiltration of CFSE green fluorescently labeled CD8^+^ T cells in orthotopic Panc02 or DT6066 pancreatic tumors following EV_DC_@Mn‐DNA treatment compared to other groups, including EV_DC_@DNA group (Figure S12A–C, Supporting Information). Recent studies suggested that the level of tumor STING activity or/and glycolysis might be correlated with vasculature remodeling.^[^ ^7^, ^40^ ^]^ To determine whether this was the case for EV_DC_@Mn‐DNA mediated tumor vascular remodeling, we analyzed the expression level of phosphorylated STING (p‐STING) and glycolysis‐related protein such as Glut1 (glucose transporter 1) around tumor blood vessels and in the tumor parenchyma through IF staining. In the EV_DC_@Mn‐DNA group, p‐STING signals were elevated around tumor blood vessels, predominantly co‐localizing with CD11c⁺ DCs rather than CD8⁺ T cells, and were higher than in other groups, including EV_DC_@DNA (Figure 6E–G and Figure S13A–C, Supporting Information). Subsequently, mice receiving EV_DC_@Mn‐DNA displayed markedly reduced Glut1 expression in perivascular tumor regions compared with other groups, including EV_DC_@DNA. This was accompanied by α‐SMA immunostaining to identify fibroblasts and Sirius Red staining to visualize collagen fibers, showing reduced fibroblast abundance and diminished collagen deposition within the parenchyma of orthotopic Panc02 and DT6066 pancreatic tumors (Figure 6H–J and Figure S13D–F, Supporting Information). Similarly, prior research has shown that remodeling of tumor vasculature can lead to a reduction in desmoplasia in pancreatic tumors.^[^ ^4^, ^6^ ^]^

To confirm in vivo uptake of EV_DC_@Mn‐DNA by intratumoral DCs, we intravenously injected FAM‐labeled Mn‐DNA, FAM‐labeled DNA, or EV_DC_ encapsulating FAM‐labeled Mn‐DNA or DNA alone (EV_DC_@(FAM)Mn‐DNA or EV_DC_@(FAM)DNA) into orthotopic Panc02 tumor‐bearing mice. The harvested tumors were digested and subjected for FACS analysis based on the gating strategy illustrated in Figure 6K. The results indicated a robust FAM signal intensity detected specifically in CD11c^+^MHC‐II^+^ DCs, while it was not observed in other immune cell types such as CD3^−^NK1.1^+^NK (natural killer) cells, CD3^+^T cells and CD11c^−^MHC‐II^+^ cells in tumors from mice that were treated with EV_DC_@(FAM)Mn‐DNA or EV_DC_@(FAM)DNA as compared to those treated with FAM labeled Mn‐DNA complex or DNA alone (Figure 6L), suggesting that the specific uptake of Mn‐DNA complexes from EV_D_c@Mn‐DNA by endogenous DCs. In summary, these findings suggest that treatment with EV_DC_@Mn‐DNA contributes to remodeling of tumor vasculature, likely through its influence on the cGAS‐STING and glycolysis related pathways. This results in improved blood vessel functionality and increased expression of endothelial‐ICAM1, which in turn reduces hypoxia and desmoplasia, together facilitating the infiltration of immune cells into the tumor parenchyma.

### 2.7 EV_DC_@Mn‐DNA Treatment Exerts Immunostimulatory Activity in Human Pancreatic Cancer

**Figure 7:**
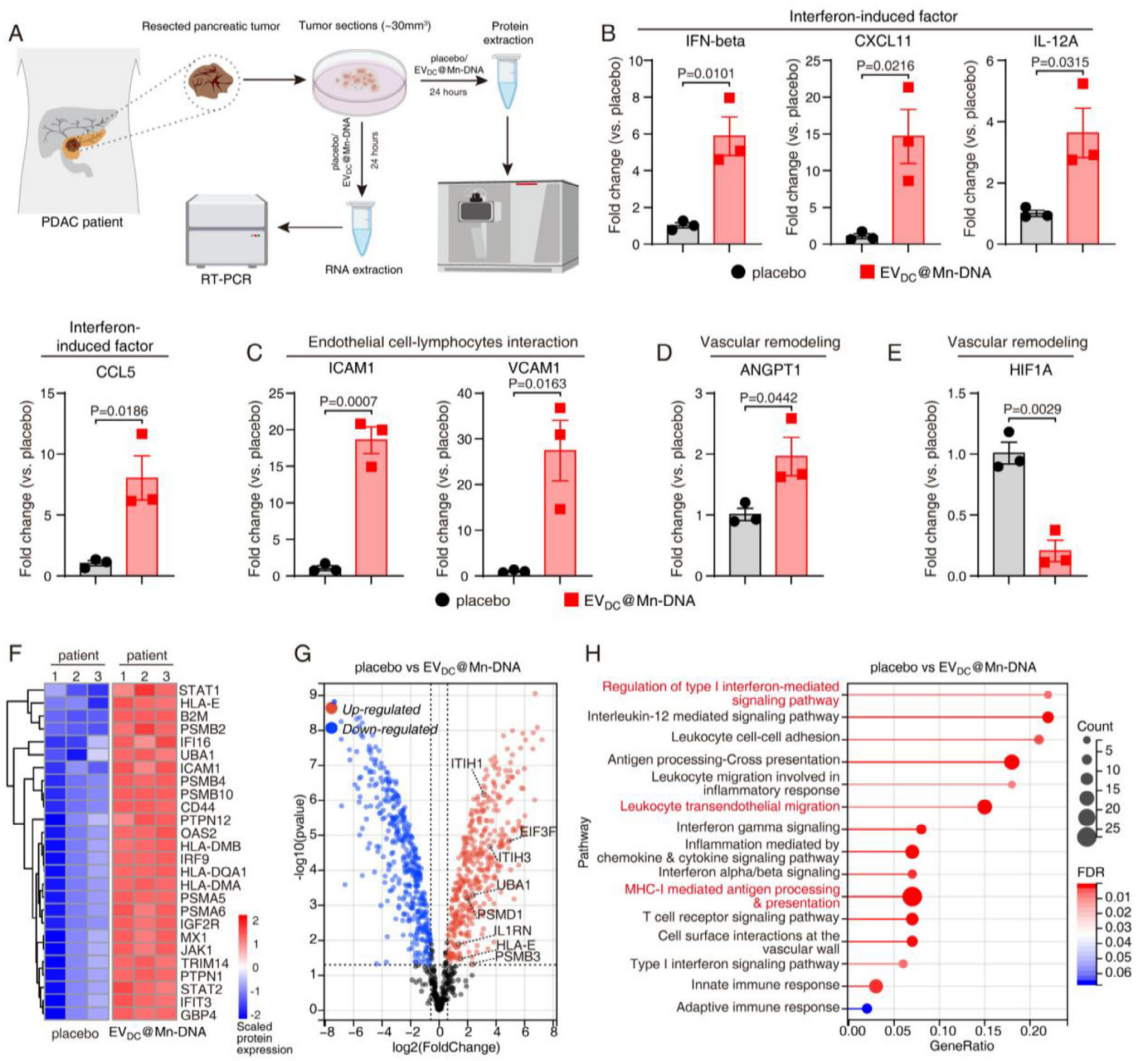
EV_DC_@Mn‐DNA treatment provokes immunostimulatory activity in human PDAC cancer. A) Schematic diagram of an ex vivo human PDAC tissue model. B–E) RT‐PCR analysis of the indicated gene expression in ex vivo human PDAC tissues after treatment with placebo or EV_DC_@Mn‐DNA (n = 3 patients per group). F) Heatmap analysis of the proteomics data of ex vivo human PDAC tissues after treatment with placebo or EV_DC_@Mn‐DNA. G) Volcano plot analysis of the proteomics data in F. The up‐regulated proteins related to the cGAS‐STING pathway are highlighted. H) KEGG enrichment pathway analysis of the proteomics data in F. The pathways associated with the immunostimulatory activity are highlighted in red. Results represent mean ± S.E.M. B–E) Unpaired two‐tailed student's *t*‐test.

To enhance the clinical relevance of our findings, we established an ex vivo model using PDAC tissues obtained from three distinct patients. These tissues were sectioned into ≈30 mm^3^ cubes and directly exposed to placebo or EV_DC_@Mn‐DNA (**Figure** **7**A). Subsequent analysis via RT‐PCR and proteomics revealed notable results. Specifically, the RT‐PCR data demonstrated an upregulation in the expression of interferon‐induced factors such as IFN‐beta, CXCL11, and IL‐12A, along with T cell recruitment‐related cytokine CCL5, endothelial cell‐immune cell interaction markers ICAM1 and VCAM1, and markers linked to vascular remodeling such as increased ANGPT1 and reduced HIF1A expression in PDAC tissues treated with EV_DC_@Mn‐DNA compared to those treated with the placebo (Figure 7B–E). Moreover, combined heatmap analysis, volcano plots, and KEGG analysis of the proteomics data demonstrated a notable enrichment in pathways related to the regulation of type 1 interferon‐mediated signaling, leukocyte transendothelial migration, MHC‐I‐mediated antigen processing and presentation, among others, in PDAC tissues treated with EV_DC_@Mn‐DNA compared to the placebo‐treated samples (Figure 7F–H). Overall, our findings validate the efficacy of EV_DC_@Mn‐DNA in PDAC tissues from three distinct patients, providing initial evidence supporting the potential utility of EV_DC_@Mn‐DNA as a strategy for coordinating anti‐tumor immunity and vascular remodeling in human PDAC.

## 3 Discussion

Our results demonstrate that Mn^2^⁺ converts tumor cell–derived DNA from B‐form to Z‐form, enhancing its binding to cGAS and promoting cGAS phase separation, thereby amplifying cGAS–STING activation in DCs. Encapsulation of Mn‐DNA within DC‐derived EVs protects it during circulation and facilitates targeted delivery to intratumoral DCs, leading to robust cGAS–STING activation. This restores vascular–immune homeostasis in immune‐cold pancreatic tumors, strengthens anti‐tumor immunity, and suppresses tumor growth more effectively than DNA alone delivered via EV_DC_. These findings highlight a promising immunotherapeutic strategy for overcoming the challenges of pancreatic cancer treatment.

Metal elements, such as manganese, play a crucial role in enhancing cellular resistance to infections by augmenting pathogen genetic material recognition through cytoplasmic DNA sensors like cGAS.^[^ ^27^ ^]^ Several studies indicated that Mn^2+^ not only activates cGAS by increasing its sensitivity to DNA and enzymatic activity but also enhances STING activity by increasing cGAMP‐STING binding affinity.^[^ ^26^ ^]^ However, it remains unclear whether Mn^2^⁺ improves DNA binding to cGAS and induces its phase separation, which is known to facilitate activation.^[^ ^18^ ^]^ Here, we provide the first evidence that Mn^2^⁺ promotes the conformational transition of DNA from the B‐form into the Z‐form. Consistently, recent studies indicated that the accumulation of Z‐form mitochondrial DNA favored for Z‐DNA binding protein 1 (ZBP1)‐mediated cGAS‐STING pathway activation.^[^ ^41^ ^]^ We demonstrated that Mn‐DNA complexes enhance cGAS phase separation, further amplifying cGAS‐STING pathway activation more effectively than DNA alone. This discovery led to the development of a cost‐effective and accessible cGAS‐STING pathway activator, laying a foundation for manganese‐based immunotherapy strategies.

Clinical research on STING agonists for cancer faces challenges due to local STING pathway inhibition, poor pharmacokinetic and physicochemical properties and limited systemic delivery methods.^[^ ^14^ ^]^ While nanoformulations enhance anti‐tumor effects,^[^ ^40^ ^]^ the indiscriminate delivery of STING agonists may trigger both anti‐tumor immunity and tumor immune tolerance.^[^ ^8^ ^]^ To overcome these challenges, selectively targeting cGAS‐STING signaling to dendritic cells,^[^ ^36^ ^]^ while avoiding impacts on immunosuppressive cells, is a potential strategy. However, there is limited research on this approach within the tumor microenvironment. In response to this challenge, DC‐derived EVs^[^ ^23^, ^42^ ^]^ have emerged as a potential carrier for delivering our Mn‐DNA complexes. Indeed, pulsing DCs with tumor cell lysates enables them to present a broad repertoire of tumor‐associated antigens, enhancing their ability to activate tumor‐specific T cells. These antigen‐pulsed DCs have been tested in preclinical and clinical settings,^[^ ^43^, ^44^, ^45^ ^]^ and therefore can serve as a safe and effective source for producing EVs for pancreatic cancer immunotherapy. Unlike tumor cell–derived EVs, which may carry malignant signals,^[^ ^46^ ^]^ tumor cell lysate pulsed DC–derived EVs are immunogenic and have been explored as tumor vaccines due to their ability to carry MHC–antigen peptide complexes and directly present antigens to T cells.^[^ ^20^ ^]^ In this study, we developed Raft‐Ultra technology to efficiently incorporate Mn‐DNA complexes into tumor cell lysate pulsed DC‐derived immunogenic EVs. This approach preserves the EVs' capacity to activate T cells and present tumor antigens while also stimulating antigen‐presenting cells. Notably, we created EV_DC_@Mn‐DNA, which delivers Mn‐DNA complexes into the cytoplasm via membrane fusion and lysosomal escape, enhancing cGAS‐STING activation and accelerating DC maturation. This delivery method also transfers the MHC‐tumor antigen peptide complex directly to DCs on the EV_DC_@Mn‐DNA surface, accelerating the host's anti‐tumor immune response. Compared to directly encapsulating STING agonists,^[^ ^14^ ^]^ EV_DC_@Mn‐DNA avoids off‐target toxicity, offers superior tumor targeting, and is more cost‐effective. Additionally, unlike prevalent chemotherapeutic drug strategies inducing DNA release,^[^ ^47^ ^]^ EV_DC_@Mn‐DNA activates the cGAS‐STING pathway with negligible cytotoxicity^[^ ^48^ ^]^ and no impact on DCs' tumor antigen presentation capacity.^[^ ^49^ ^]^ While systemic Mn^2^⁺ administration remains impractical for clinical applications,^[^ ^50^ ^]^ encapsulating Mn‐DNA complexes in DC‐derived immunogenic EVs provides a viable alternative and is far more effective than EVs carrying DNA alone.

Transforming the immunosuppressive tumor microenvironment into one conducive to immune response remains an intractable challenge in current cancer therapeutics.^[^ ^3^ ^]^ Our EV_DC_@Mn‐DNA treatment selectively activates STING signaling in intratumoral DCs, effectively remodeling the immunosuppressive tumor microenvironment in pancreatic cancer. This results in increased activation of DCs—particularly the cDC1 subset, which is essential for anti‐tumor immunity^[^ ^51^ ^]^—expansion of T cell populations, enlarged tertiary lymphoid structures, and the conversion of immunologically cold tumors into hot phenotypes. Indeed, recent studies have shown that the number of activated DCs determine pancreatic tumor immune status and response to immunotherapy.^[^ ^52^ ^]^ Additionally, we show that targeting and activating the cGAS‐STING signaling pathway in perivascular DCs by EV_DC_@Mn‐DNA may re‐program tumor metabolism to improve blood vessel perfusion, reduce hypoxia and enhance drug delivery, a phenotype known as vasculature remodeling.^[^ ^4^, ^5^, ^6^, ^7^ ^]^ Additionally, we show that EV_DC_@Mn‐DNA treatment enhances ICAM1 expression on vascular endothelial cells, which is known to involve in facilitating immune cell adhesion and infiltration.^[^ ^12^, ^53^ ^]^ Importantly, we provided strong evidence confirming the specific uptake of EV_DC_@Mn‐DNA and its DNA by intratumor DCs in orthotopic pancreatic tumor models. Finally, the use of ex vivo human PDAC models confirmed the clinical significance of our EV_DC_@Mn‐DNA approach. In conclusion, this study provides insights into using a DC specific cGAS activator EV_DC_@Mn‐DNA to beneficially modulate the vascular‐immune homeostasis in pancreatic tumors, offering a potent immunotherapy approach.

This study has certain limitations. Although we demonstrate that Mn^2^⁺ induces tumor cell DNA to adopt the Z‐DNA conformation and enhances cGAS–STING activation, the precise mechanism underlying this conformational switch remains unclear and will require further structural and biophysical investigation. In addition, while promising results were obtained in murine and ex vivo human models, broader evaluation of safety, efficacy across diverse tumor types and immune contexts, and the scalability of the DC‐derived EV delivery system will be critical for clinical translation. Addressing these limitations will enable refinement of delivery strategies, deeper mechanistic insight, and validation of therapeutic potential in preclinical and clinical settings.

## 4 Experimental Section

### Tissue Culture

Mouse dendritic cell line (DC2.4) was acquired from Millipore (Cat#SCC142M), while the mouse pancreatic tumor cell line DT6066 was generously provided by Professor Kairbaan Hodivala‐Dilke. For the choice of cell models, DC2.4 has been widely used to investigate dendritic cell functions,^[^ ^20^, ^32^ ^]^ while DT6066, derived from murine pancreatic cancer, has been extensively employed in both in vitro and in vivo studies.^[^ ^6^, ^30^ ^]^ Mouse pancreatic cancer cell line Panc02 was purchased from National Collection of Authenticated Cell Cultures (Cat#SCSP‐5468). Human pancreatic cancer cell line Panc1 was purchased from American type culture collection (Cat#CRL‐1469). These cell lines were cultured in RPMI1640 (GIBCO, Cat#C11875500BT)/DMEM (Cat#C11995500BT, GIBCO) medium containing glucose 4500 mg L^−1^, L‐Glutamine 584.0 mg L^−1^,1% penicillin‐streptomycin (Cat#15 070 063, GIBCO) and 10% fetal bovine serum (FBS) (Cat#10270‐106, GIBCO) in 37 °C and 5% CO_2_ incubator. After cell recovery, the cells were passaged for at least three stable generations before being used in experiments.

### DNA Extraction, Modification, and Coordination

DNA was extracted from DT6066, Panc02 and Panc1 cancer cells using the GeneJET genomic DNA purification kit (Cat#K0721, ThermoFisher Scientific) following the manufacturer's instructions. Then the purified tumor cell‐derived DNA was fragmented using an ultrasonic probe (SONICS, VCX 130) (100 s). Briefly, sonication was performed using a 2 mm probe at a power of 90 W, with a DNA solution (1 mL, 100 µg mL^−1^) placed in an ice bath. The sonication cycle was set to 5 s on and 5 s off, for a total duration of 100 s. DNA fragments were subsequently purified using a DNA purification kit (Cat#DP204‐03, TIANGEN). The molecular weight of DNA following sonication was evaluated by agarose gel electrophoresis (2% agarose gel, 120 V at room temperature for 30 min), and the bands were visualized using a gel imaging system (ChampGel 6000, SINSAGE, China). Gel electrophoresis analysis showed that the fragments ranged from 200 to 1000 bp. To ensure complete modification in subsequent procedures, the molar concentration of DNA fragments was calculated based on a fragment length of 200 bp. Biotinylation of DNA fragments was conducted a Biotin 3′ end DNA labeling kit (Cat#D3106, Beyotime) according to the manufacturer's instructions. Briefly, DNA fragments (1 µM) were incubated with terminal deoxynucleotidyl transferase (10 U µL^−1^) and Biotin‐11‐dUTP (5 µM) at 37 °C for 30 min. The resulting biotinylated DNA was purified using a DNA purification kit (Cat#DP204‐03, TIANGEN). The DNA:biotin ratio was 1:1.

### Coordination of Mn^2+^ and DNA

Mn^2+^ was incubated with tumor cell (DT6066, Panc02 or Panc1)‐derived DNA (based on the number of nucleotide base pairs) at a molar ratio of 1:1, 1:5, 1:10, 1:20, or 1:50 for 4 h under stirring (MMS4pro, JOANLAB, China, 800 rpm min^−1^). To remove residual free Mn^2+^, the reaction mixture was loaded into a dialysis bag (Cat#D6191, Sigma‐Aldrich, molecular weight cutoff, 50 kDa) and dialyzed against DNase‐free double‐distilled water at a 1:100 volume ratio. The external solution was refreshed every 4 h for six cycles, after which the Mn‐DNA complexes were collected by lyophilization. The Mn‐DNA complexes were redissolved in saline to a final concentration of 20 µg mL^−1^. To quantify Mn^2^⁺ content in DNA, samples were digested in concentrated nitric acid (65%) at room temperature for 30 min, followed by heating at 90 °C for 2 h on a hot plate in a fume hood until complete dissolution was achieved. After cooling, the digested samples were diluted 50‐fold with ultrapure water and filtered through a 0.22 µm (Millipore, SLGPR33RB) membrane to remove insoluble particles. The Mn^2^⁺ concentration was then measured using inductively coupled plasma atomic emission spectroscopy (ICP‐AES) (OPtima8300, PerkinElmer) according to the manufacturer's instruction. The ICP‐AES analysis was performed using an instrument operating at an RF incident power of 1300 W. The plasma argon gas flow rate was maintained at 12 L min^−1^, and the nebulizer gas flow rate was set to 0.55 L min^−1^. Prior to sample analysis, the instrument was calibrated using a series of standard solutions with known concentrations of the target elements. A blank solution was used to correct for background signals, and the calibration curve was constructed based on the linear relationship between emission intensity and analyte concentration. All measurements were performed in triplicate to ensure accuracy and reproducibility.

### Production and Purification of Recombinant cGAS Protein

Escherichia coli BL21 (DE3) cells (1 × 10⁸ cells) were transfected with the 1–2 µg of pET28a vector (Novagen) containing the full‐length mouse cGAS gene sequence with a C‐terminal His tag. The cells were cultured in LB medium supplemented with kanamycin until reaching an OD600 of 1.0. Subsequently, isopropyl‐β‐D‐thiogalactopyranoside (IPTG) was added to a final concentration of 0.4 mM to induce protein expression. For cultivation, 200 mL of inoculated medium was transferred into a 500 mL autoclaved Erlenmeyer flask and incubated in a shaking incubator (ZS‐CR, HUAYUAN) at 25 °C with an agitation speed of 200 rpm for an additional 12 h. The bacteria were harvested by centrifugation at 3000×g at 4 °C for 10 min (centrifuge type (Sorvall ST4R Plus, ThermoFisher Scientific), rotor specifications (TX‐1000, ThermoFisher Scientific) and then lysed using lysozyme and lysis buffer (Cat#P2229S‐2, Beyotime) to extract total protein. The His‐tagged cGAS protein was subsequently purified using a His‐tag protein purification kit (Cat#P2229S, Beyotime) following the manufacturer's instructions. GFP‐cGAS protein overexpression vector was purchased from Guangzhou IGE Biotechnology LTD.

### Quantification of DNA‐cGAS Binding through Bio‐Layer Interferometry

Bio‐layer interferometry (BLI) assays were conducted using ForteBio's Octet RED96 system following the manufacturer's protocols. Briefly, Octet streptavidin (SA) biosensors (Cat#18‐5020, Sartorus) were equilibrated in phosphate‐buffered saline (PBS, 10 mM) containing 0.05% Tween 20 (PBST) for 10 min at room temperature before each assay. The sensors were then loaded with biotinylated DNA or Mn‐DNA complexes at varying concentrations for 5 min. Equilibrium dissociation constants (KD) were determined from the binding data obtained with recombinant His‐tagged cGAS protein interacting with DNA alone or Mn‐DNA complexes. Data analysis was performed using Octet Data Analysis software (Sartorus). In some experiments, the affinity of Mn‐DNA complexes with different coordination ratios and recombinant His‐tagged cGAS was assessed using BLI to identify the optimal coordination molar ratios between DNA and Mn^2+^ ions.

### Phase Separation Assay

To investigate the interaction between cGAS and Mn‐DNA/DNA alone, in vitro phase separation assays were performed. First, Cy3‐labeled DNA was synthesized via an acylation reaction. Briefly, DNA fragments (1 µM) were incubated with terminal deoxynucleotidyl transferase (TdT) (Cat#EP0162, ThermoFisher Scientific, 10 U µL^−1^) and 5‐aminoallyl‐dUTP (Cat#AM8439, ThermoFisher Scientific, 5 µM) at 37 °C for 30 min. The resulting amino‐modified DNA was purified using a DNA purification kit (Cat#DP204‐03, TIANGEN). The purified DNA (1 µM) was then reacted with Cy3‐NHS (Cat#HY‐112498, MCE, 50 µM) in 0.1 M sodium bicarbonate buffer (pH8.5) at room temperature in the dark for 2 h. Finally, the Cy3‐labeled DNA was purified using a DNA purification kit (Cat#DP204‐03, TIANGEN). Recombinant GFP‐cGAS protein (final concentration: 10 µM) and Cy3‐labeled Mn‐DNA/DNA alone (final concentration: 10 µM) were mixed in a phase separation buffer containing 10% PEG8000, 2 mM DTT, 5 mM MgCl_2_, and 150 mM NaCl in Tris‐HCl (pH 7.4). The reaction mixture (total volume: 10 µL) was incubated at 37 °C for 30 min, after which 10 µL was applied onto a glass slide and imaged using a confocal fluorescence microscope (Leica, SP8 STED 3X) with 40× microscope objective (Leica, HC PL APO 40×/1.30 OIL).

### Fluorescence Recovery after Photobleaching Assay

To assess the dynamic properties of phase‐separated droplets formed by cGAS and Mn‐DNA/DNA alone, FRAP experiments were performed. Phase‐separated droplets were positioned in the center of the confocal microscope field of view, and the central region of each droplet was photobleached using a 488 nm laser. Fluorescence recovery was monitored by capturing images at 3‐s intervals over a 200‐s period. Fluorescence intensity was quantified using ImageJ software.

### Circular Dichroism Spectroscopy

To determine the effect of Mn^2^⁺ coordination on DNA conformation, CD spectroscopy was performed. Mn‐DNA was prepared in phosphate buffer (final concentration: 10 nM) and analyzed using a CD spectrometer (Applied Photophysics Chirascan Plus, Applied Photophysics Ltd.) in a quartz cuvette. Spectra were recorded in the wavelength range of 200–320 nm at room temperature.

### Nuclear Magnetic Resonance Phosphorus Spectroscopy

Mn‐DNA was dissolved in phosphate buffer (50 mM, pH 8.0) containing 10% deuterium oxide. Phosphorus (P) signals in DNA were analyzed using a 500 MHz NMR spectrometer (AVIII500HD, BRUKER). Spectra were processed using TopSpin software.

### Immunofluorescence Staining

Dendritic cells (DCs) (5 × 10^5^ cell) were seeded in 20 mm glass bottom cell culture dish (Cat#801 001, NEST) and cultured overnight until reaching ≈70% confluence. Cells were transfected with Mn‐DNA using Lipofectamine 3000 (Cat#L3000015, ThermoFisher Scientific) and incubated for 6 h. The transfection procedure was performed as follows: Mn‐DNA (containing 2500 ng of DNA) was diluted in 125 µL of Opti‐MEM (Cat#31 985 070, ThermoFisher Scientific) to prepare the DNA mixture. Separately, 5 µL of Lipofectamine 3000 was diluted in another 125 µL of Opti‐MEM to prepare the lipid mixture. The two mixtures were then combined, gently mixed, and incubated at room temperature for 15 min. The resulting transfection complex was added to DCs, which were subsequently incubated at 37 °C for 6 h in a cell culture incubator. Following transfection, cells were washed three times with PBS and fixed with 4% paraformaldehyde at room temperature for 10 min. After PBS washes, cells were permeabilized with 0.1% Triton X‐100 (Cat#9036‐19‐5, Sigma‐Aldrich, 1 mg mL^−1^) for 5 min and blocked with 3% BSA (Cat#9048‐46‐8, Sigma‐Aldrich, 30 mg mL^−1^) for 1 h at room temperature. Primary antibodies (rabbit mAb against mouse cGAS (Cat#31 659, Cell signaling technology, 1:50), mouse mAb against zDNA (Cat#ab00783‐3.3, BIOZOL, 1:50)) were incubated at room temperature for 1 h, followed by PBS washes and incubation with fluorescent dye‐conjugated secondary antibodies for 1 h. After additional PBS washes, glass slides were mounted using DAPI‐containing mounting medium (Cat#ZLI‐9557, ZSGB‐BIO) and imaged using a confocal fluorescence microscope (Leica, SP8 STED 3X) with 40× microscope objective (Leica, HC PL APO 40×/1.30 OIL). To quantitatively assess phase separation in DCs, we counted the number of cGAS condensate droplets per field of view across 100 cells, with three fields collected per group and the experiment repeated independently three times for statistical analysis.

### Biotinylated DNA Pull‐Down

The DNA pull‐down experiment followed established protocols with some modifications.^[^ ^54^ ^]^ Briefly, purified recombinant mouse His‐tagged cGAS protein (500 nM) and Mn^2+^ ions (20 nM), along with biotinylated DNA (100 nM) or biotinylated Mn‐DNA complexes (100 nM), were co‐incubated in 100 µL of Pierce IP lysis buffer (Cat#87 787, ThermoFisher Scientific) at 4 °C for 2 h. Subsequently, 20 µL of streptavidin‐coated magnetic beads (Cat#P2151, Beyotime; ≈200 nm in diameter, 10 mg mL^−1^ bead concentration, with over 6 mg mL^−1^ streptavidin) were added to the mixture and incubated at 4 °C for 2 h. The beads were then collected using a magnetic rack and washed with IP lysis buffer. The His‐cGAS bound to the magnetic beads was isolated for subsequent western blotting. Additionally, 2.5% of the input and empty magnetic beads were loaded onto the same gel as a reference and negative control, respectively, during western blot analysis.

### Generation and Characterization of EV_DC_@Mn‐DNA

Human/mouse dendritic cells (DC) derived EVs (EV_hDC_/EV_DC_) were prepared following established procedures with some modifications,^[^ ^20^ ^]^ and the manganese‐human/mouse tumor cell derived DNA complexes (Mn‐DNA complexes) were packaged into EV_hDC_ or EV_DC_ using our newly developed Raft‐Ultra method. Briefly, human/mouse cancer cell lysates containing tumor antigens were obtained through freeze‐thaw cycles and incubated with human DCs/mouse DC2.4 cells for 12 h in the presence of 1 µg mL^−1^ lipopolysaccharide (LPS). After discarding the culturing medium, human DCs/mouse DC2.4 cells were exposed to UV irradiation (×100 µJ cm^−2^) and cultured in a serum‐free medium for another 24 h. The cell debris in the supernatant was then removed through the centrifugation step (3214×g at 4 °C for 30 min), and the EV_hDC_/EV_DC_ was collected using a high‐speed centrifuge to work at 20 802×g at 4 °C for 60 min. Afterward, the EV_hDC_/EV_DC_ was dispersed in saline (20 mg protein mL^−1^) for subsequent experiments. To prepare EV_hDC_@Mn‐DNA/EV_DC_@Mn‐DNA, EV_hDC_/EV_DC_ (20 mg protein mL^−1^) was mixed with Mn‐DNA complexes by vortexing, then frozen quickly in liquid nitrogen for 10 min, and reconstituted at 37 °C water bath, repeated three times, and then the solution was sonicated for 15 min. After centrifugation (20 802×g at 4 °C for 60 min) to remove the free Mn‐DNA complexes, EV_hDC_@Mn‐DNA/EV_DC_@Mn‐DNA was obtained and dispersed in saline (5 mg protein mL^−1^) for subsequent experiments.

The morphology of EV_DC_@Mn‐DNA was examined using transmission electron microscopy (Tecnai G2 SpiritTwin, FEI). For the sample preparation, original EV_DC,_ Raft‐Ultra method treated EV_DC_ or EV_DC_@Mn‐DNA was added dropwise to a copper grid, absorbed for 2 min, followed by staining with a 2% phosphotungstic acid solution (G‐CLONE, Cat#RS2930) for 30 s, which were then washed with deionized water three times. Afterward, the morphology of EV_DC_@Mn‐DNA was observed after the copper grid was installed in the transmission electron microscopy instrument. For confocal fluorescent imaging analysis, Dil‐labeled EV_DC_@Mn‐(FAM)DNA was first prepared. Briefly, FAM‐labeled DNA was synthesized via an acylation reaction. DNA fragments (1 µM) were incubated with TdT (Cat#EP0162, ThermoFisher Scientific, 10 U µL^−1^) and 5‐aminoallyl‐dUTP (Cat#AM8439, ThermoFisher Scientific, 5 µM) at 37 °C for 30 min. The resulting amino‐modified DNA was purified using a DNA purification kit (Cat#DP204‐03, TIANGEN). The purified DNA (1 µM) was then reacted with 5/6‐FAM‐SE (Cat#46 409, ThermoFisher Scientific, 50 µM) in 0.1 M sodium bicarbonate buffer (pH8.5) at room temperature in the dark for 2 h. FAM‐labeled DNA was subsequently purified using the same DNA purification kit and coordination with Mn^2+^ to form Mn‐(FAM)DNA and encapsulated into EV_DC_, which was further incubated with Dil (Cat#C7001, ThermoFisher Scientific, 5 µM) at 37 °C in the dark for 1 h. Free Dil was removed by centrifugation at 20 802×g at 4 °C for 1 h in the dark, followed by three washes with PBS, the Dil labeled EV_DC_@Mn‐(FAM)DNA was obtained. EV_DC_@Mn‐(FAM)DNA was mounted on glass slides with coverslips and examined by confocal fluorescence microscope (Leica, SP8 STED 3X) with 40× microscope objective (Leica, HC PL APO 40×/1.30 OIL).

### Nanoparticle Tracking Analysis

The diameter of EV_DC_@Mn‐DNA was measured using nanoparticle tracking analysis (NTA) (NanoSight NS300, Malvern). Briefly, EV_DC_@Mn‐DNA was diluted in saline to achieve an optimal concentration (≈3 × 10⁸ particles mL^−1^) and loaded into the sample chamber using a syringe pump. For each sample, five 60‐s videos were recorded at a camera level of 15, with a detection threshold set to 5. The analysis was carried out using NTA software version 3.4, and the viscosity of saline was set at 0.99Cp. All measurements were conducted at room temperature (25 °C), and particle size were calculated as the mean of five independent recordings.

### Atomic Force Microscope Analysis

The morphology of EV_DC_@Mn‐DNA was detected by atomic force microscopy (AFM, SPM9700, Shimadzu). Briefly, EV_DC_@Mn‐DNA was diluted with deionized water to 10 ng protein mL^−1^ and coated on the surface of silicon wafer to detect the morphology. First, EV_DC_@Mn‐DNA was diluted with deionized water to a final concentration of 10 ng protein mL^−1^. Then, 100 µL of the diluted solution was dropped onto the surface of a silicon wafer and allowed to adsorb for 40 min at room temperature. After adsorption, the surface was gently rinsed three times with 200 µL of deionized water and air‐dried naturally. The sample was then subjected to AFM to analyze its morphology. Samples were imaged using the NanoScope software with a scan size of 600 nm × 600 nm, a scan rate of 1.00 Hz, and a resolution of 256 samples per line. The scan angle was set to 0.00°, with both X and Y offsets at 0.000 nm. The feedback gain was 5.721, and the peak force setpoint was 0.006375 V. The system operated with a peak force amplitude of 150 nm and a peak force frequency of 2 kHz. The lift height during scanning was 300 nm. A ScanAsyst‐Air probe with a spring constant of 0.4 N m^−1^ was used as the cantilever.

### Cellular Uptake Assay

To examine the cellular uptake behavior of EV_DC_@Mn‐DNA by DC2.4 cells, Dil‐labeled EV_DC_@Mn‐(FAM)DNA was first prepared. Briefly, FAM‐labeled DNA was synthesized via an acylation reaction. DNA fragments (1 µM) were incubated with TdT (Cat#EP0162, ThermoFisher Scientific, 10 U µL^−1^) and 5‐aminoallyl‐dUTP (Cat#AM8439, ThermoFisher Scientific, 5 µM) at 37 °C for 30 min. The resulting amino‐modified DNA was purified using a DNA purification kit (Cat#DP204‐03, TIANGEN). The purified DNA (1 µM) was then reacted with 5/6‐FAM‐SE (Cat#46 409, ThermoFisher Scientific, 50 µM) in 0.1 M sodium bicarbonate buffer (pH8.5) at room temperature in the dark for 2 h. FAM‐labeled DNA was subsequently purified using the same DNA purification kit. EV_DC_@Mn‐(FAM)DNA (10 µg protein mL^−1^) was prepared according to the above protocol and incubated with Dil (Cat#C7001, ThermoFisher Scientific, 5 µM) at 37 °C in the dark for 1 h. Free Dil was removed by centrifugation at 20 802×g in the dark for 1 h at 4 °C, followed by three washes with PBS, the Dil labeled EV_DC_@Mn‐(FAM)DNA was obtained. DC2.4 cells were incubated with fluorescein‐modified EV_DC_@Mn‐DNA (200 ng DNA mL^−1^ and 10 µg protein mL^−1^) for 0.5, 2, 4, 6, and 8 h. After discarding the culturing medium and washing with PBS, DCs were washed three times with PBS and fixed with 4% paraformaldehyde at room temperature for 10 min. After PBS washes, cells were permeabilized with 0.1% Triton X‐100 (Cat#9036‐19‐5, Sigma‐Aldrich, 1 mg mL^−1^) for 5 min and blocked with 3% BSA (Cat#9048‐46‐8, Sigma‐Aldrich, 30 mg mL^−1^) for 1 h at room temperature. fluorescent dye‐conjugated primary antibodies (Alexa Fluor 488 conjugated anti‐LAMP1 antibody (Cat#58996S, Cell signaling technology, 1:50) were incubated at room temperature for 1 h. After additional PBS washes, glass slides were mounted using DAPI‐containing mounting medium (Cat#ZLI‐9557, ZSGB‐BIO) and imaged using a confocal fluorescence microscope (Leica, SP8 STED 3X) with 40× microscope objective (Leica, HC PL APO 40×/1.30 OIL). In some experiments, after Dil labeled EV_DC_@Mn‐DNA treatment for 6 h, DC2.4 cells were stained with DiO (Cat#D5840, Solarbio, 5 µM) and Alexa Fluor 488 conjugated anti‐LAMP1 antibody (Cat#58996S, Cell signaling technology, 1:50). Fluorescence images were analyzed using ImageJ 1.52 V. The uptake of Dil‐labeled EV_DC_@Mn‐(FAM)DNA by DC2.4 cells was assessed using flow cytometry. Briefly, cells were incubated with Dil‐labeled EV_DC_@Mn‐(FAM)DNA for 0.5, 2, 4, 6, and 8 h. After incubation, the culture medium was discarded, and cells were washed three times with PBS. DC2.4 cells were then detached using trypsin and collected by centrifugation at 500×g for 5 min at 4 °C. Fluorescence intensity was measured using a flow cytometer (CytoFLEX S, Beckman, USA), and data were analyzed with FlowJo V10.

### Immunofluorescence and Immunohistochemistry Analysis

Immunofluorescence and immunohistochemistry staining was performed on the tumor sections as described previously.^[^ ^55^ ^]^ The antibodies used for IHC and IF staining included: rabbit mAb against mouse cGAS (Cat#31 659; Cell signaling technology, 1:200) mouse mAb against Z‐DNA/Z‐RNA (Cat#ab00783‐3.3, BIOZOL, 1:50), rabbit mAb against human/mouse anti‐Ki‐67 (Cat#GB111141, Servicebio, 1:1000), rabbit mAb against human/mouse CD34 (Cat#ab81289, Abcam, 1:300), rat mAb against mouse endomucin (Cat#sc‐65495, Santa Cruz Biotechnology, 1:100), mouse mAb against human/mouse α‐smooth muscle actin (α‐SMA)‐Cy3TM antibody (Cat#C6198, Sigma‐Aldrich, 1:500), rabbit mAb against human/mouse Glut1 (Abcam, Cat#ab652, 1:100), rabbit mAb against mouse CD3 (Cat#78 588, Cell signaling technology, 1:200), rabbit mAb against mouse CD4 (Cat#48 274, Cell signaling technology, 1:200), rabbit mAb against mouse CD8 (Cat#98 941, Cell signaling technology, 1:200), rabbit mAb against mouse CD20 (Cat#70 168, Cell signaling technology, 1:200), rabbit mAb against mouse CD11c (Cat#97 585, Cell signaling technology, 1:200), rabbit mAb against mouse CD86 (Cat#91 882, Cell signaling technology, 1:200), rabbit mAb against human/mouse p‐STING (Cat#72 971, Cell signaling technology, 1:200), rabbit mAb against human/mouse CCL5 (Cat#36 467, Cell signaling technology, 1:200), rabbit mAb against human/mouse CXCL9 (Cat#ab202961, Abcam, 1:200), rabbit mAb against mouse granzyme B (Cat#44 153, Cell signaling technology, 1:200), rabbit mAb against mouse perforin (Cat#31 647, Cell signaling technology, 1:200). Antigen retrieval was performed under high pressure conditions for 10 min using the unmasking buffer (Tris‐HCL, pH = 9.2). The staining intensity was calculated from five different fields using ImageJ software. In some experiments, DC2.4 cells pretreated with the specified treatments in the figures were fixed with formalin and permeabilized cell membrane using Triton‐X100 (Cat#T8787, Sigma‐Aldrich) and subsequently immunostained with primary antibodies including rabbit mAb against human/mouse p‐STING (Cat#72 971, Cell signaling technology, 1:200) and p‐IRF3 (Cat#29 047, Cell signaling technology, 1:200).

### Western Blotting

Protein extracts of EV_DC_@Mn‐DNA and DC2.4 cells after treatments were prepared by utilizing RIPA lysis buffer (Cat#P0013B, Beyotime) supplemented with 1% protease inhibitors (Cat#MSSAFE‐5VL‐17407262, Sigma Aldrich). The protein concentration in each extract was measured using the Pierce BCA Protein Assay Kits (Cat#23 227, ThermoFisher Scientific). Subsequently, 50 µg protein from each sample was loaded onto sodium dodecyl sulfate‐polyacrylamide gels (10% gel) for electrophoresis. Electrophoresis was carried out at 80 V for 30 min, then switched to 120 V for another 30 min. Follow by transferring the proteins onto a nitrocellulose membrane at 100 V for 2 h in an ice‐water bath, the membrane was incubated with 5% BSA solution in PBS buffer with 0.1% Tween‐20 (PBS‐T) at room temperature for 2 h. Then, the primary antibodies, which diluted in PBS‐T containing 3% BSA at a ratio of 1:1000 (v/v), were added to the membranes and incubated for 12 h at 4 °C. Following second antibody incubation, chemiluminescence signals were detected using the Mini Chemiluminescent Imaging and Analysis System (MiniChemi 610, Sage Creation Science). The following primary antibodies were used: anti‐β‐actin antibody (Cat#sc‐47778, Santa Cruz, 1:5000), anti‐p‐TBK1 antibody (Cat#5483S, Cell signaling technology, 1:1000), anti‐TBK1 antibody (Cat#3504S, Cell signaling technology, 1:1000), anti‐p‐STING antibody (Cat#72971S, Cell signaling technology, 1:1000), anti‐STING antibody (Cat#13647S, Cell signaling technology, 1:1000), anti‐TSG101 antibody (Cat#ab125011, Abcam, 1:1000), anti‐Calnexin antibody (Cat#AF5362, Affinity Biosciences, 1:1000) and anti‐CD63 antibody (Cat#NBP2‐36567, Novus, 1:1000). In certain experiments, DC2.4 were stimulated with EV_DC_@Mn‐DNA pretreated with DNase (Cat#10 104 159 001, Roche, 10 U mL^−1^), or EV_DC_@Mn‐DNA first treated with Triton‐X100 (Cat#T8787, Sigma‐Aldrich, 1 mg mL^−1^) to rupture the membrane and then treated with DNase (Cat#10 104 159 001, Roche, 10 U mL^−1^).

### Sample Preparation and Analysis for Proteomics

The preparation and analysis of proteomics were preformed according to established protocols as described previously.^[^ ^7^ ^]^ Initially, protein extraction from DC2.4 cells which pretreated with saline and EV_DC_@Mn‐DNA were prepared using RIPA lysis buffer supplemented with 1% protease inhibitor cocktail (Cat#PPC1010‐5ML, Sigma‐Aldrich). Subsequently, the protein extracts were precipitated using quadruple volume acetone. Then the dried protein precipitates were redissolved in urea solution (8 M). Followed by reduction using 2 mM dithiothreitol (DTT) for 1.5 h, and alkylation using 12 mM iodoacetamide (IAA) in dark for another 45 min, the proteins was digested overnight using Pierce Trypsin Protease, MS Grade (Cat#90 057, ThermoFisher Scientific) with a final concentration of 0.02 µg µL^−1^. Then the digestion process was terminated with trifluoroacetic acid (TFA). Desalting of the resulting peptide solution was performed using a reversed‐phase C18 spin column. Subsequently, the dried peptides were desolved in TFA solution (0.1%) and analyzed by LC‐MS/MS using the ThermoFisher Scientific^s^ Orbitrap Fusion instrument following the manufacturer's instructions. The obtained raw data files were processed using Proteome Discoverer (ThermoFisher Scientific‐v2.4) for label‐free quantification (LFQ) and searched against the UniProt mouse database. The above‐described sample preparation and analysis procedures for proteomics were also applied to placebo‐ or EV_DC_@Mn‐DNA‐ treated human PDAC tissues.

### Quantitative Real‐Time PCR Analysis

For the qRT‐PCR analysis using DC2.4 cells, the cells were seeded into 6‐well plates at a density of 6 × 10^5^ cells per well and cultured overnight. When the cells reached ≈80% confluence, 6 µL of Lipofectamine 3000 (Cat#L3000015, ThermoFisher Scientific) was mixed with 6 µL of each treatment (placebo (saline; 0.9% w/v NaCl solution), Mn^2+^, DNA alone, or Mn‐DNA complexes) in 250 µL of Opti‐MEM (Cat#31 985 070, ThermoFisher Scientific). The mixtures were incubated at room temperature for 15 min and subsequently added to the DC2.4 cells, yielding final concentrations of 0.6 µM Mn^2+^ and 3 µM DNA, respectively. The cells were incubated at 37 °C in cell incubator containing 5% CO_2_ for 4 h, then lysed using Trizol (Cat#15 596 018, Invitrogen), composed primarily of phenol and guanidine isothiocyanate, to extract RNA, which was subsequently transcribed into cDNA using TransSctipt Uni All‐in‐One First‐Strand cDNA Synthesis SuperMix for qPCR (Cat#AT341‐01, Transgen). Following this, cDNA (20 ng), primers (0.2 µmol L^−1^), and 10 µL SYBR Premix Ex Taq (Cat#RR820A, TaKaRa) were mixed, and qRT‐PCR analysis was performed using the Roche LightCycler 480 instrument as per the manufacturer's instructions. Analysis of the raw data was conducted using LightCycler 480 Software Version 1.5.1. For the qRT‐PCR analysis of human PDAC tissues, we initially treated the tumor tissues with either placebo or EV_DC_@Mn‐DNA for 48 h. Subsequently, the tissues were lysed with Trizol to extract RNA for cDNA synthesis, following the method described earlier. All primer sequences utilized in this analysis were procured from Gene WIZ, and their details are accessible in Table S1, Supporting Information.

### Protocol for Treating Orthotopic Pancreatic Cancer Models

Orthotopic pancreatic tumor model was established and monitored following previously described protocols^[^ ^30^ ^]^ and the instructions provided by the ultrasound imaging manufacturer (VisualSonics). Male C57BL/6 mice (4‐ to 6‐week‐old) were injected with 30 µL (1 PBS:1 Matrigel) of 5 × 10^5^ DT6066 or 5 × 10^5^ Panc02 cells into pancreas. 14 days post‐injection (Day 14), the pancreatic tumor‐bearing C57BL/6 mice were randomly divided into six groups (n = 5 per group) and treated with placebo (100 µL saline per mouse), EV_DC_ (intravenous dose of 10 mg protein kg^−1^ per mouse) in 100 µL saline, DNA plus EV_DC_ (intravenous dose of 200 µg DNA kg^−1^ (DNA alone) + 10 mg protein kg^−1^ (EV_DC_) per mouse) in 100 µL saline, Mn‐DNA plus EV_DC_ (intravenous dose of 200 µg DNA kg^−1^ (Mn‐DNA complexes) + 10 mg protein kg^−1^ (EV_DC_) per mouse) in 100 µL saline, EV_DC_@DNA (intravenous dose of 200 µg DNA kg^−1^ (Encapsulated DNA alone) and 10 mg protein kg^−1^ per mouse) in 100 µL saline or EV_DC_@Mn‐DNA (intravenous dose of 200 µg DNA kg^−1^ (Encapsulated Mn‐DNA complexes) and 10 mg protein kg^−1^ per mouse) in 100 µL saline via intravenous injection once every 5 days for a total of 3 treatments. Meanwhile, the tumor growth was monitored using the VisualSonics Vevo2100 system with a MS400 transducer every 5 days for a period of 20 days, with body weight recorded concurrently. At the experimental endpoint (Day 34) (10 days after the final treatment), mice were euthanized, and tumors, tumor‐draining lymph nodes, and major organs—including the heart, liver, spleen, lungs, and kidneys—were collected for immunostaining, immunohistochemistry, histopathology and FACS analysis. Mice were anesthetized with isoflurane during the experiments. Isoflurane anesthesia parameters in mice: For ultrasound imaging, induction was performed with 3–4% isoflurane, followed by maintenance at 1.0–1.5% with O_2_ at 0.5–0.8 L min^−1^.

For in vivo fluorescein‐labeled anti‐PD‐L1 tracking experiment, we first treated orthotopic DT6066 or Panc02 pancreatic tumor bearing mice with either placebo or EV_DC_@Mn‐DNA every 5 days for three times, were given a third treatment 6 h before intravenous injection of 50 µL of fluorescein‐labeled anti‐PD‐L1 (Cat#568 304, BD biosciences). 1‐ h post‐injection, the mice first received an intravenous administration of 20 µg of anti‐PE‐PECAM antibody (PE‐conjugated, Cat#102 408, Biolegend). Soon after, orthotopic pancreatic tumors were subjected to confocal microscopy imaging using a 25*×* water immersion objective (Olympus, NA 1.05) over 45 min. Z‐stack images were acquired across 70 slices, encompassing a depth of up to 100 µm. The imaging setup included a tunable pulsed chameleon infrared multiphoton laser (MaiTai eHODS and INSIGHT X3) and PMT (GaAsp). During imaging, the mice were anesthetized using isoflurane gas anesthesia. Isoflurane anesthesia parameters in mice: For optical imaging, induction was performed with 3–4% isoflurane, followed by maintenance at 1.0–1.5% with O_2_ at 0.5–0.8 L min^−1^.

For in vivo CD3^+^CD8^+^ T cell tracking experiment, the CD3^+^CD8^+^ T cells were first stained with green fluorescent dye carboxyfluorescein diacetate succinimidyl ester (CFSE, Cat#C34554, ThermoFisher Scientific, 5 µM) for 10 min at room temperature following the manufacturer's instruction. Mice bearing orthotopic pancreatic tumors were first treated with placebo, EV_DC_, DNA+EV_DC_, Mn‐DNA+EV_DC_, EV_DC_@DNA or EV_DC_@Mn‐DNA every 5 days for three times, were given a third treatment 6 h before intravenous injection of 1 × 10^6^ CFSE labeled CD3^+^CD8^+^ T cells and subsequently injected with anti‐PE‐PECAM antibody intravenously 1 h later. Finally, the mice were subjected to multi‐photon confocal imaging as described before. Mice were anesthetized with isoflurane during the experiments. Isoflurane anesthesia parameters in mice: For optical imaging, induction was performed with 3–4% isoflurane, followed by maintenance at 1.0–1.5% with O_2_ at 0.5–0.8 L min^−1^.

To assess tumor blood vessel functions, orthotopic pancreatic tumor‐bearing mice were initially treated with either placebo, EV_DC_, DNA+EV_DC_, Mn‐DNA+EV_DC_, EV_DC_@DNA or EV_DC_@Mn‐DNA for three times. They received a third treatment 6 h prior to intravenous (IV) injection of 20 µg of FITC‐conjugated ICAM1 antibody (Cat#116 105, Biolegend) followed by IV injection of 20 µg of anti‐PE‐PECAM antibody (Cat#102 408, Biolegend) 1 h later. Finally, the orthotopic pancreatic tumors were subjected to in vivo multi‐photon confocal imaging as described above. Mice were anesthetized with isoflurane during the experiments. Isoflurane anesthesia parameters in mice: For optimal imaging, induction was performed with 3–4% isoflurane, followed by maintenance at 1.0–1.5% with O_2_ at 0.5–0.8 L min^−1^.

### Tumor Blood Perfusion and Flow Analysis

The blood perfusion and flow status of orthotopic pancreatic tumors were evaluated using the Vevo 2100 system (VisualSonics) following the manufacturer's instructions. Briefly, mice with orthotopic pancreatic tumors, treated with either placebo, EV_DC_, DNA+EV_DC_, Mn‐DNA+EV_DC_, EV_DC_@DNA or EV_DC_@Mn‐DNA every 5 days for up to three treatments, received an intravenous injection of 100 µL of Vevo Micromarker suspension (VisualSonics). Subsequently, the blood perfusion and flow in the entire tumor or tumor core sites were monitored using the VevoCQ contrast quantification software. Mice were anesthetized with isoflurane during the experiments. Isoflurane anesthesia parameters in mice: For ultrasound imaging, induction was performed with 3–4% isoflurane, followed by maintenance at 1.0–1.5% with O_2_ at 0.5–0.8 L min^−1^.

### Biodistribution of EV_DC_@Mn‐DNA

The biodistribution of EV_DC_@Mn‐DNA was assessed based on a previously published method with some modifications.^[^ ^56^ ^]^ EV_DC_@Mn‐DNA were labeled with the near‐infrared dye DiR (1,1′‐dioctadecyl‐3,3,3′‐dio‐tetramethylindotricarbocyanine iodide) and evaluated using a near‐infrared fluorescent imaging system (IVIS Spectrum, PerkinElmer). For DiR labeling, EV_DC_@Mn‐DNA (20 mg protein mL^−1^) was incubated with DiR (Cat#51306‐35‐5, Ruixibio, 1 mg mL^−1^) at 37 °C for 1 h in the dark, followed by centrifugation (20 802×g at 4 °C for 1 h in the dark) to collect the DiR‐labeled EV_DC_@Mn‐DNA. The pellet was washed three times with PBS to remove unbound dye. For tumor implantation, 1 × 10^6^ DT6066 tumor cells in 100 µL were subcutaneously injected into the lower left flank of male C57BL/6 mice (4–6 weeks old). When tumors reached a volume of 200–300 mm^3^, mice received intravenous injections of either DiR‐labeled EV_DC_@Mn‐DNA (10 mg protein kg; DiR: 0.5 mg kg^−1^) or free DiR (0.5 mg kg^−1^). The near‐infrared fluorescent signal in the mice was monitored using the imaging system at 1, 4, 8, and 12 h post‐intravenous injection. At the 12‐h time point, the mice were euthanized, and tumors as well as major organs (heart, liver, spleen, lungs, and kidneys) were harvested for ex vivo imaging. Data were analyzed using imaging analysis software (Caliper Life Sciences).

### Flow Cytometry (FACS) Analysis

Immune cells from tumors were isolated using the Mouse Lymphocyte Separation Medium (Cat#7 211 011, Dakewe) following the manufacturer's instructions. After blocking with CD16/32 (Cat#101 302, Biolegend, 1 µg per 10^6^ cells in 100 µL) for 30 min at 25 °C, the isolated immune cells were incubated with primary antibodies in the dark for 45 min at 4 °C. The cells were then washed three times with PBS and resuspended in FACS buffer (PBS without Ca^2+^ and Mg^2+^, 2% FBS, 5 mM EDTA). For intracellular cytokine/transcription factors staining, intracellular fixation and permeabilization buffer (eBioscience) were used. Briefly, the immune cells were fixed at 25 °C for 20 min, washed with PBS to remove the fixation buffer, then suspended in permeabilization buffer and incubated with primary antibodies at 4 °C for 30 min. After a repeated washing process, the cells were resuspended in FACS buffer and analyzed using flow cytometry (CytoFLEX S, Beckman, USA) with FlowJo V10 software. For the FACS analysis, the following primary antibodies were used: anti‐mouse BV421‐CD11c (Cat#117 343, Biolegend, 0.25 µg per 10^6^ cells in 100 µL), anti‐mouse APC‐CD86 (Cat#105 045, Biolegend, 0.125 µg per 10^6^ cells in 100 µL), anti‐mouse BV510‐CD3 (Cat#100 233, Biolegend, 0.5 µg per 10^6^ cells in 100 µL), anti‐mouse AF750‐CD4 (Cat#100 459, Biolegend, 0.25 µg per 10^6^ cells in 100 µL), anti‐mouse PE‐CD8 (Cat#100 707, Biolegend, 0.25 µg per 10^6^ cells in 100 µL), anti‐mouse APC‐CD69 (Cat#104 513, Biolegend, 1 µg per 10^6^ cells in 100 µL), anti‐human/mouse FITC‐Ki‐67 (Cat#151 211, Biolegend, 0.5 µg per 10^6^ cells in 100 µL), anti‐mouse BV421‐CD62L (Cat#104 435, Biolegend, 0.125 µg per 10^6^ cells in 100 µL), anti‐mouse PE/Cyanine7‐CD25 (Cat#102 015, Biolegend, 1 µg per 10^6^ cells in 100 µL), anti‐mouse/rat/human PE‐FOXP3 (Cat#320 007, Biolegend, 5 µL per 10^6^ cells in 100 µL), anti‐mouse APC/Fire 750‐CD45 (Cat#103 153, Biolegend, 0.25 µg per 10^6^ cells in 100 µL), anti‐mouse PE/Cyanine7‐CD40 (Cat#124 621, Biolegend, 1 µg per 10^6^ cells in 100 µL), anti‐mouse PE‐CD80 (Cat#104 707, Biolegend, 0.5 µg per 10^6^ cells in 100 µL), anti‐mouse APC‐I‐A/I‐E (Cat#107 614, Biolegend, 0.25 µg per 10^6^ cells in 100 µL), anti‐mouse PE‐NK1.1 (Cat#156 503, Biolegend, 0.25 µg per 10^6^ cells in 100 µL) and anti‐mouse APC‐IFNy (Cat#505 810, Biolegend, 1 µg per 10^6^ cells in 100 µL).

The proliferation of CD8^+^ T cells was assessed using the CFSE kit (Cat#C34554, ThermoFisher Scientific, 5 µM) as per the manufacturer's instructions. The mouse CD8^+^ T cells from spleen were labeled with CFSE (5 µM) in serum‐free PBS in the dark for 10 min, washed with PBS, and then cultured for 72 h. Cells were cultured at 37 °C in a humidified incubator with 5% CO_2_ using RPMI 1640 medium supplemented with 10% fetal bovine serum (FBS) and 10 ng mL^−1^ IL‐2. For experimental treatments, cells were assigned to different groups and exposed to the indicated reagents: placebo (saline), CD3/CD28 [anti‐mouse CD3 antibody (Cat#100 340, Biolegend, 5 µg mL^−1^) and anti‐mouse CD28 antibody (Cat#102 116, Biolegend, 5 µg mL^−1^], EV_DC_ (10 µg protein mL^−1^), DNA (200 ng DNA mL^−1^) + EV_DC_ (10 µg protein mL^−1^), EV_DC_@DNA (200 ng DNA mL^−1^ and 10 µg protein mL^−1^), Mn‐DNA (200 ng DNA mL^−1^) + EV_DC_ (10 µg protein mL^−1^), or EV_DC_@Mn‐DNA (200 ng DNA mL^−1^ and 10 µg protein mL^−1^). The CFSE‐labeled CD8^+^ T cells were then subjected to FACS analysis.

### Dendritic Cell Maturation Experiments

Mouse bone marrow‐derived DC (BMDC) were prepared as described previously.^[^ ^20^ ^]^ Briefly, bone marrow cells were isolated from the femurs and tibias of healthy male C57BL/6 mice (4–6 weeks old), filtered, and subjected to RBC lysis. Cells were seeded at 1 × 10⁶ per well in 24‐well plates and cultured for 7 days in complete RPMI‐1640 medium supplemented with 10% FBS and GM‐CSF (40 ng mL^−1^) to generate BMDCs. BMDCs (1 × 10^6^ cells well^−1^) were seeded in 24‐well plates, and treatments were applied immediately upon seeding. Cells were exposed to either placebo (saline), lipopolysaccharide (LPS) (1 µg mL^−1^), EV_DC_ (10 µg protein mL^−1^), DNA + EV_DC_ (200 ng DNA mL^−1^ and 10 µg protein mL^−1^), EV_DC_@DNA (200 ng DNA mL^−1^ and 10 µg protein mL^−1^), Mn‐DNA + EV_DC_ (200 ng DNA mL^−1^ and 10 µg protein mL^−1^), or EV_DC_@Mn‐DNA (200 ng DNA mL^−1^ and 10 µg protein mL^−1^). After 24 h of incubation, the cell suspensions were collected, centrifuged at 500 g for 5 min, and resuspended in 200 µL PBS for further analysis. Then cells were incubated with primary antibodies for 45 min at 4 °C, washed with PBS, resuspended in FACS buffer, and subjected to flow cytometry (CytoFLEX S, Beckman, USA) analysis, with data analyzed using FlowJo V10. For human dendritic cell maturation experiments, human DCs were derived from CD14^+^ monocytes obtained from the peripheral blood of healthy individuals using ficoll density gradient centrifugation. Briefly, 9 mL Ficoll (Cat#BL590, Biosharp; density: 1.077 g mL^−1^) was added into a 50‐mL centrifuge tube, and 15 mL of peripheral blood was carefully layered onto the Ficoll. The samples were centrifuged at 450×g for 25 min, after which the peripheral blood mononuclear cells (PBMCs) layer was collected and resuspended in 30 mL PBS. PBMCs were harvested by centrifugation at 500×g for 5 min, washed once with PBS, and subsequently resuspended in PBS, and CD14 positive monocytes were then purified from PBMCs using the human CD14 microbeads ultrapure kit (Cat#130‐118‐906, Miltenyi). Immature human DCs were subsequently cultured in RPMI 1640 medium supplemented with 10% FBS, 1% penicillin‐streptomycin, GM‐CSF (Cat#300‐03, PeproTech, 40 ng mL^−1^), and IL‐4 (Cat#200‐04, PeproTech, 40 ng mL^−1^) at 37 °C in a humidified incubator with 5% CO_2_ for 7 days, and then subjected to placebo, lipopolysaccharide (LPS) (Cat#L2880, Sigma‐Aldrich, 1 µg mL^−1^), EV_hDC_ (10 µg protein mL^−1^), DNA (200 ng DNA mL^−1^) + EV_hDC_ (10 µg protein mL^−1^), EV_hDC_@DNA (200 ng DNA mL^−1^ and 10 µg protein mL^−1^), Mn‐DNA (200 ng DNA mL^−1^) + EV_hDC_ (10 µg protein mL^−1^), or EV_hDC_@Mn‐DNA (200 ng DNA mL^−1^ and 10 µg protein mL^−1^) treatment for 24 h.

### T Cell Experiments

The mouse T cells were isolated from spleen lymphocytes using a pan T cell isolation kit (Cat#130‐095‐130 Miltenyi for mouse). The isolated T cells (1 × 10^6^ cells well^−1^) were seeded in 24‐well plates. Cells were collected, incubated with primary antibodies for 45 min at 4 °C, washed with PBS, resuspended in FACS buffer, and analyzed via flow cytometry (CytoFLEX S, Beckman, USA), with data analyzed using FlowJo V10.

### OVA‐Tetramer Experiment

The process of EV_DC_ presenting antigen to T cells was conducted using T‐Select H‐2K^b^ OVA Tetramer‐SIINFEKL‐PE (Cat#TS‐5001‐1C, MBL). OVA‐specific CD8^+^ T cells were identified using OVA (H‐2K^b^/SIINFEKL) tetramers. This assay is based on the principle that MHC class I molecules loaded with the SIINFEKL peptide form tetramers that bind with high avidity to the T cell receptors of antigen‐specific CD8^+^ T cells, allowing their precise detection by flow cytometry. Briefly, mouse DCs were then incubated with SIINFEKL peptide (10 µg mL^−1^) or control peptide (PSVHSYILVTALGIT) for 6 h at 37 °C. After three washes with PBS, the DCs were stimulated with ultraviolet irradiation (×100 µJ cm^−2^) and cultured in serum‐free medium for 24 h, and the EV_SIINFEKL‐DC_ or EV_control peptide‐DC_ were collected by centrifugation. Mn‐DNA complexes were then packed into these EV_DC_ via the Raft‐Ultra method as described above. The mouse T cells (1 × 10^6^ cells well^−1^) from spleen were seeded in a 24‐well plate and co‐cultured with or without control peptide (10 µg protein mL^−1^), OVA epitope peptide (10 µg protein mL^−1^), original EV_SIINFEKL‐DC_/EV_control peptide‐DC_, Raft‐Ultra treated EV_SIINFEKL‐DC_/EV_control peptide‐DC_ or EV_SIINFEKL‐DC_@Mn‐DNA/EV_control peptide‐DC_@Mn‐DNA for 12 h. After three washes with PBS, T cells were resuspended in FACS buffer. Subsequently, cells were stained with T‐Select H‐2K^b^ OVA Tetramer‐SIINFEKL‐PE (Cat#TS‐5001‐1C, MBL, 0.25 µg per 10^6^ cells in 100 µL), anti‐mouse BV510‐CD3 (Cat#100 233, Biolegend, 0.5 µg per 10^6^ cells in 100 µL) and anti‐mouse FITC‐CD8 (Cat#140 403, Biolegend, 0.25 µg per 10^6^ cells in 100 µL) at 4 °C in the dark for 45 min, washed with PBS, and resuspended in FACS buffer. Flow cytometry (CytoFLEX S, Beckman, USA) was used for sample detection, and the data were analyzed using FlowJo V10. For the DC membrane fusion experiment, immature DCs were pre‐treated with EV_SIINFEKL‐DC_@Mn‐DNA for 6 h at 37 °C to collect mature DCs. Mouse T cells (1 × 10^6^ cells well^−1^) were seeded in a 24‐well plate and co‐cultured with or without SIINFEKL peptide, EV_DC_@Mn‐DNA, mature DCs (pretreated with EV_SIINFEKL‐DC_@Mn‐DNA) in the presence or absence of 10 µM STING inhibitor C‐176 (Cat#S6575, Selleck).

### In Vivo Uptake Experiment of EV_DC_@Mn‐DNA in Tumors

The in vivo uptake study of EV_DC_@Mn‐DNA in tumors was performed by using flow cytometry technique following the manufacturer's protocol. Orthotopic pancreatic tumor model was established and monitored following previously described protocols.^[^ ^30^ ^]^ Male C57BL/6 mice (4‐ to 6‐week‐old) were injected with 30 µL (1 PBS:1 Matrigel) of 5 × 10^5^ Panc02 cancer cells into pancreas. 14 days post‐injection, FAM‐labeled DNA (200 µg DNA kg^−1^ per mouse), FAM‐labeled Mn‐DNA complexes (200 µg DNA kg^−1^ per mouse), EV_DC_ encapsulating FAM‐labeled DNA (200 µg DNA kg^−1^ and 10 mg protein kg^−1^ per mouse) or EV_DC_ encapsulating FAM‐labeled Mn‐DNA complexes (200 µg DNA kg^−1^ and 10 mg protein kg^−1^ per mouse) were intravenously administered to mice bearing orthotopic pancreatic tumors. 8 h post‐injection, the mice were euthanized, and their tumors were dissected and cut into 3 cubic millimeter sections. These tissue sections underwent collagenase III digestion for 1 h at 37 °C to obtain a single‐cell suspension. The digested material was filtered through a 70 µm strainer, and the filtrates were centrifuged at 500×g for 5 min to collect cell pellets. The cells were resuspended in 3 mL lymphocyte separation buffer (Cat#7 211 011, Dakewe), overlaid with 500 µL RPMI 1640 medium, and centrifuged at 800×g for 30 min. The lymphocyte layer was carefully collected, transferred into 10 mL RPMI 1640 medium, and centrifuged at 500×g for 5 min. Finally, the lymphocytes were resuspended in 200 µL PBS for further analysis. After blocking with CD16/32 (Cat#101 302, Biolegend, 1 µg per 10^6^ cells in 100 µL) for 30 min at 25 °C, the isolated immune cells were incubated with primary antibodies targeting T cell, NK cell and DC markers in the dark for 45 min at 4 °C. The cells were then washed three times with PBS and resuspended in FACS buffer. Flow cytometry (CytoFLEX S, Beckman, USA) analysis was conducted using FlowJo V10 to detect and analyze the samples.

### Establishment of an Ex Vivo Human PDAC Model

We obtained clinical samples from PDAC patients undergoing surgical procedures at Sun Yat‐sen Memorial Hospital, with ethical approval. Participants provided informed consent by signing a consent form. Primary PDAC tumors were submerged in RPMI 1640 with 10% FBS and cut into ≈30 mm^3^ sections using a scalpel. Each section was plated into a 12‐well plate with 2 mL of culture medium, and treatment with either placebo or EV_DC_@Mn‐DNA (10 µg protein mL^−1^ per well) was applied immediately upon seeding. After 24 h, the samples underwent RT‐PCR and proteomics analysis.

### Statistical Analysis

Statistical analysis was performed using GraphPad Prism software. Data presentations were done as mean ± Standard Error of the Mean, (S.E.M.). Two‐tailed student's *t*‐test and One/Two‐way ANOVA followed by Tukey's post hoc testing were employed for the analysis. Further details regarding the specific tests used and sample sizes (n) can be found in the figure legends. All in vitro experiments were independently repeated at least three times, unless otherwise specified in the figure legends.

### Study Approval

All animal procedures in this study were conducted in accordance with the approved guidelines provided by the Animal Ethical and Welfare Committee of Sun Yat‐sen Memorial Hospital (AP20230264). All PDAC samples were collected with written informed consent and approved by the Ethnical Review Committee of Sun Yat‐sen Memorial Hospital in Guangzhou, China (SYSKY‐2024‐487‐01).

## Conflict of Interest

The authors declare no competing interests.

## Author Contributions

X.J., L.S., X.C., and X.K. conceived and designed the experiments, analyzed and interpreted the data, co‐wrote the manuscript, and contributed equally to the paper. X.J. and L.S. carried out the generation and characterization of EVs. X.C. and X.K. performed DNA binding assays and assisted with flow cytometry experiments and analysis. X.W. and S.D. conducted proteomics data analysis. L.J., H.X., and Y.Z. provided technical advice. X.J. and L.S. carried out animal experiments. C.H. assisted with animal experiments and provided the funding. R.Z., M.W., and H.L. provided clinical samples. P.‐P.W. provided funding, conceived the study, supervised the research, and wrote the manuscript.

## Supporting information



Supporting Information

## Data Availability

The data that support the findings of this study are available from the corresponding author upon reasonable request.
