# Supplementary material for: Tailoring Vascular‐Immune Homeostasis via Manganese‐DNA Complex‐Armed Immunogenic Extracellular Vesicles for Pancreatic Cancer Immunotherapy
Source: Adv Sci (Weinh). 2025 Oct 22;13(35):e07159. doi: 10.1002/advs.202507159 (PMC13292222; doi:10.1002/advs.202507159)
Supplement: Supplementary file 1 — Supporting Information [file ADVS-13-e07159-s001.pdf]

Supporting Information

Tailoring vascular-immune homeostasis via manganese-DNA complex-armed immunogenic extracellular vesicles for pancreatic cancer immunotherapy

Xue Jiang[1,2#], Lihuan Shang[1,2#], Xiaochun Chen[1,2#], Xiangzhan Kong[1,2#], Xiaojuan Wang[1,2], Linjia Jiang[1,2], Huiying Zhao[1,2], Yafeng Zhu[1,2], Cheng Huang[1,2], Shiyi Deng[1,2], Rui Zhang[1,3*], Minghui Wang[1,4*], Haoming Lin[1,3*], Ping-Pui Wong[1,2*]

[1]Guangdong Provincial Key Laboratory of Malignant Tumor Epigenetics and Gene Regulation, Guangdong-Hong Kong Joint Laboratory for RNA medicine, Sun Yat-sen Memorial Hospital, State Key Laboratory of Oncology in South China, Sun Yat-sen University, Guangzhou, China 510120.

[2]Medical Research Center, Sun Yat-sen Memorial Hospital, Sun Yat-sen University, Guangzhou, China 510120.

[3]Guangzhou Key Laboratory of Precise Diagnosis and Treatment of Biliary Tract Cancer, Department of Biliary-Pancreatic Surgery, Sun Yat-sen Memorial Hospital, Sun Yat-sen University, Guangzhou, China 510120.

[4]Department of Thoracic surgery, Sun Yat-sen Memorial Hospital, Sun Yat-sen University, Guangzhou, China 510120.

[#]These authors contributed equally

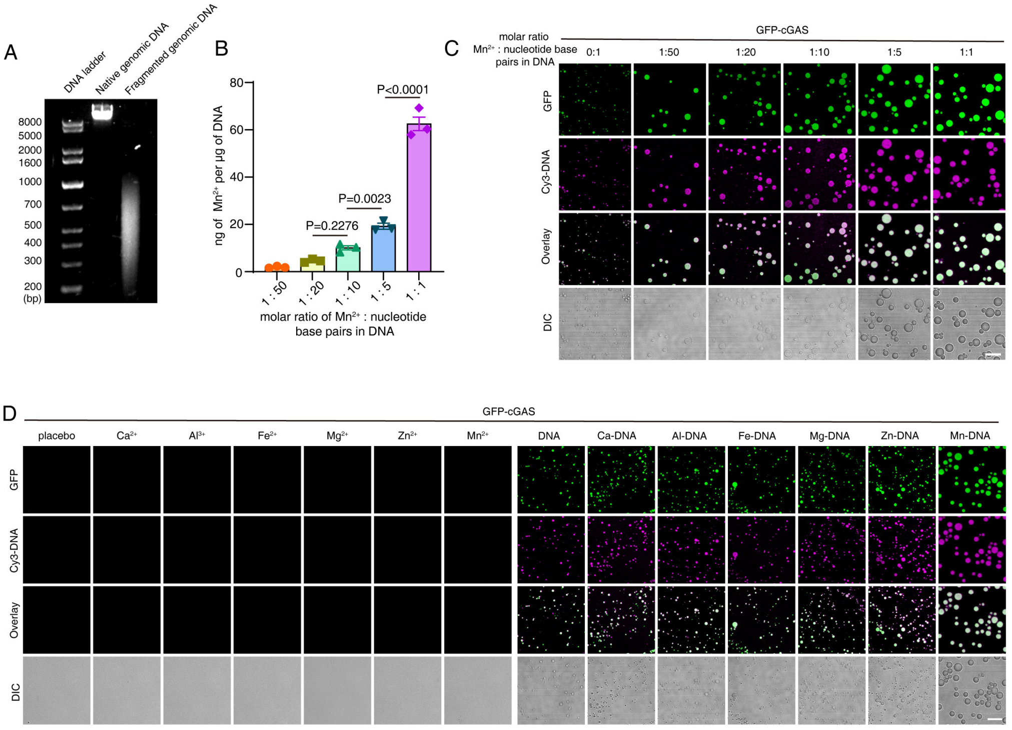

Figure S1. Mn-DNA complex treatment effectively induces cGAS phase condensation. (**A**) Agarose gel electrophoresis of native and sonication-fragmented genomic DNA from the mouse pancreatic cancer cell line DT6066, showing DNA fragments ranging from 200 to 1000 bp. A DNA ladder is included. (**B**) Bar chart depicts the amount of Mn^{2+} (ng) per µg of DNA in each group (n=3 independent experiments). (**C**) Phase separation experiments in vitro between cGAS protein and complexes formed by tumor cell-DNA with varying ratios of Mn^{2+}. (**D**) Phase separation experiments in vitro between cGAS protein and complexes formed by different metal ions with tumor cell-DNA. Results represent mean ± S.E.M. (**B**) One-way ANOVA with Tukey's post hoc test. Scale bars in (**C**, **D**) represent 10 µm.

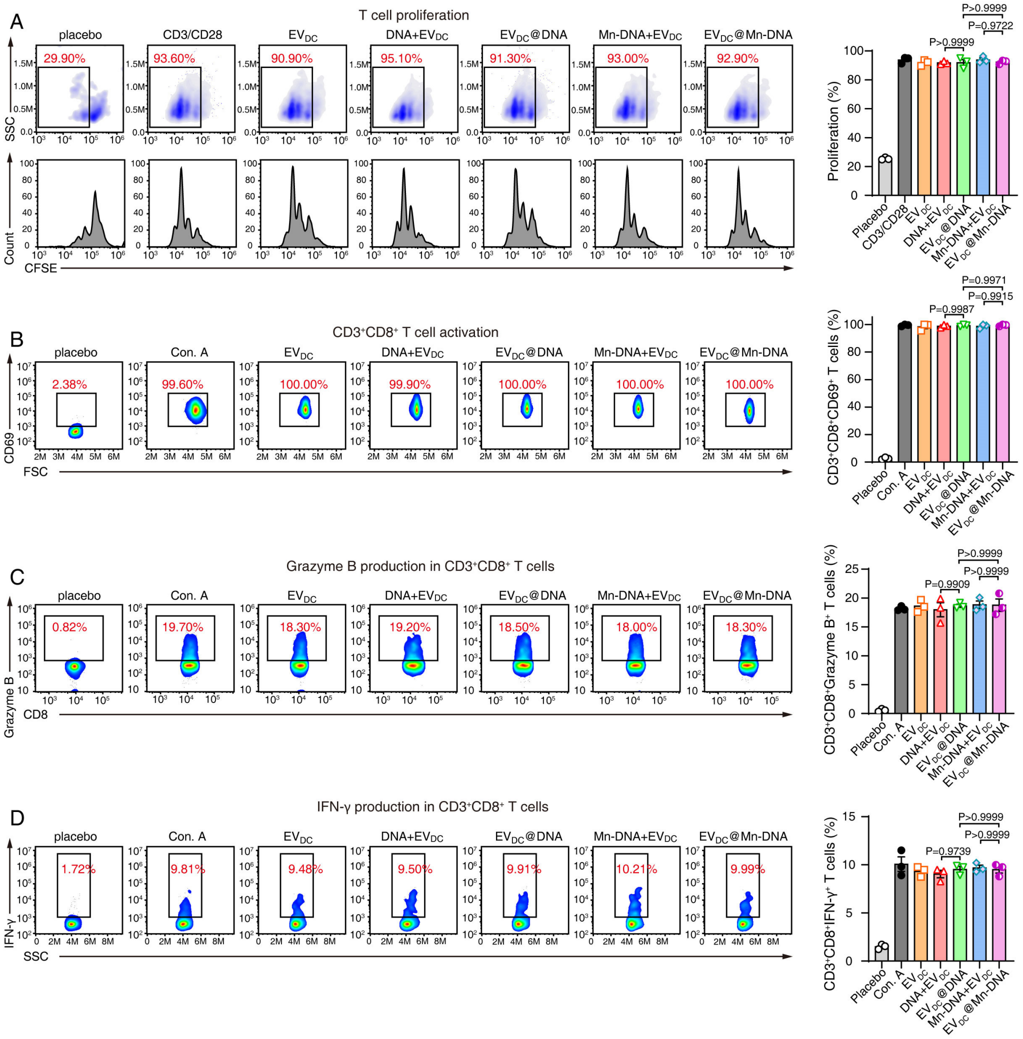

Figure S2. EV$_{DC}$@Mn-DNA treatment induces CD8$^+$ T cell proliferation and activation. (**A**) FACS analysis of CD3$^+$CD8$^+$ T cell proliferation in each group. Bar chart shows the percentage of proliferative CD3$^+$CD8$^+$ T cells in the total CD3$^+$CD8$^+$ T cell population in each group. CD3/CD28 antibodies were used as a positive control. (**B**) FACS analysis of the expression of T cell activation marker CD69 in CD3$^+$CD8$^+$ T cells in each group. Bar chart shows the percentage of CD3$^+$CD8$^+$CD69$^+$ T cells in the total CD3$^+$CD8$^+$ T cell population in each group (n= 3 independent experiments). Con. A was used as a positive control. (**C, D**) FACS analysis of the expression of granzyme

B or IFN-γ in $CD3^+CD8^+$ T cells in each group. Bar chart shows the percentage of $CD3^+CD8^+$Granzyme B^+ T cells or $CD3^+CD8^+$IFN-γ^+ in the total $CD3^+CD8^+$ T cell population in each group (n= 3 independent experiments). Results represent mean ± S.E.M. (**A-D**) One-way ANOVA with Tukey's post hoc test.

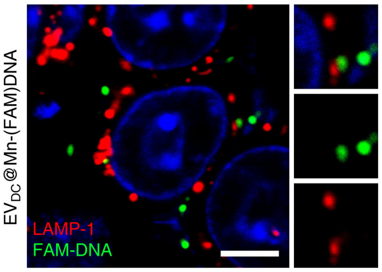

Figure S3. Mn-DNA complexes delivered to DCs by EV_{DC} escapes lysosomes. Representative fluorescent images of LAMP-1 and FAM-labeled Mn-DNA complexes in DCs treated with EV_{DC}@Mn-(FAM)DNA. Scale bars represent 10 µm.

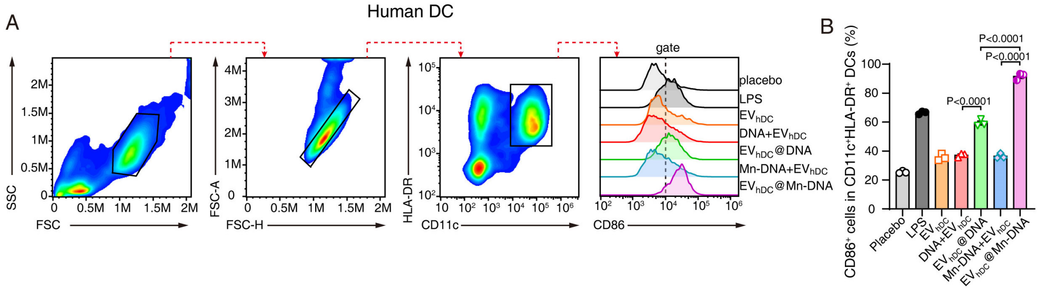

Figure S4. EV_{hDC}@Mn-DNA treatment increases human DC maturation. (**A**) FACS analysis of CD86 expression in human DCs after treated with the indicated treatments. LPS was used as a positive control. (**B**) Bar chart shows the percentage of $CD86^+$ DCs in the total population of $CD11c^+HLA\text{-}DR^+$ DCs in each group (n=3 independent experiments). Results represent mean ± S.E.M. (**B**) One-way ANOVA with Tukey's post hoc test.

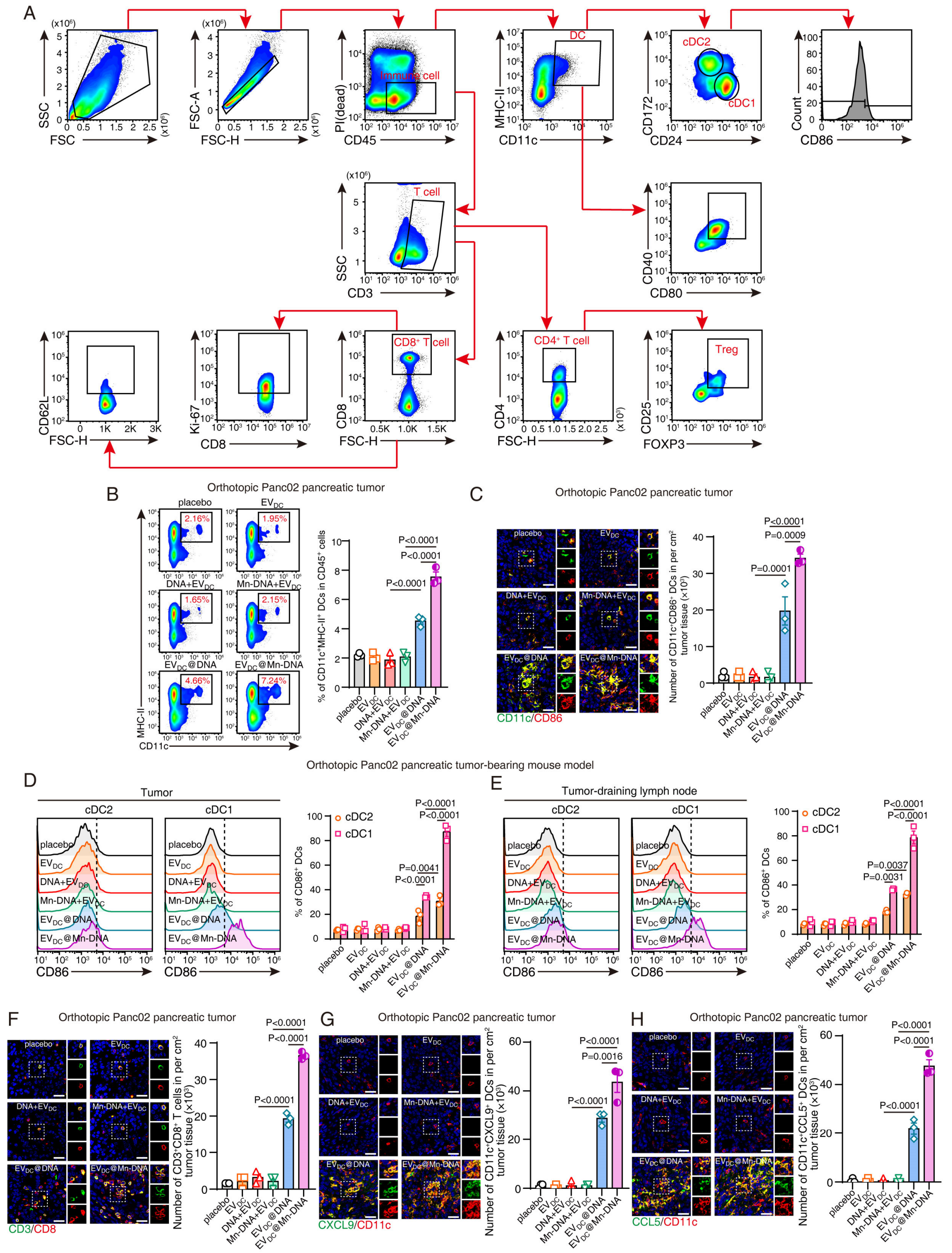

Figure S5. Administration of EV$_{DC}$@Mn-DNA reshapes the tumor immune microenvironment in orthotopic Panc02 pancreatic tumor-bearing mice. (A) The gating strategy for immune cell subsets is shown. Cells were first gated on FSC/SSC to

exclude debris and on FSC-H/FSC-A to isolate singlets. Live $CD45^+$ immune cells were defined using PI/CD45. DCs were gated from live $CD45^+$ cells on MHC-II/CD11c; cDC1 and cDC2 subsets were distinguished by CD172/CD24, and CD86 expression was evaluated within each subset. Activated DCs were identified as $CD40^+CD80^+$ populations. For Treg analysis, $CD3^+$ T cells were gated from live $CD45^+$ cells, $CD4^+$ cells were selected, and FOXP3/CD25 staining was used to define Tregs. For $CD8^+$ T cell analysis, $CD8^+$ T cells were gated from $CD3^+$ cells, and proliferation or memory status was assessed by Ki-67 or CD62L expression, respectively. (**B**) FACS analysis of CD11c and MHC-II expression in $CD45^+$ DCs from orthotopic Panc02 pancreatic tumors following the indicated treatments. Bar chart shows the percentage of $CD11c^+MHC\text{-}II^+$ DCs in the total $CD45^+$ DC population in each group (n= 3 mice per group). (**C**) Representative images of CD86 and CD11c co-immunostaining on Panc02 tumor sections derived from each group are given. Bar chart shows the number of $CD11c^+CD86^+$ DCs $\times10^3/cm^2$ in each group (n= 3 mice per group). (**D, E**) FACS analysis was performed to quantify the proportion of $CD86^+$ DCs within the cDC1 and cDC2 subsets in paired tumors and tumor-draining lymph nodes after treatment. The bar charts present the percentages of $CD86^+$ DCs within each subset across all treatment groups in both tumors and lymph nodes. (**F**) Representative images of CD3 and CD8 co-immunostaining on tumor sections derived from each group are given. Bar chart shows the number of $CD3^+CD8^+$ T cells $\times10^3/cm^2$ in each group (n= 3 mice per group). (**G, H**) Representative images of CXCL9/CCL5 and CD11c co-immunostaining on tumor sections derived from each group are given. Bar chart shows the number of

CD11c$^+$CXCL9$^+$/CCL5$^+$ DCs $\times 10^3$/cm^2 in each group (n= 3 mice per group). Results represent mean ± S.E.M. (**B**, **C**, **F-H**) One-way ANOVA with Tukey's post hoc test. (**D**, **E**) Two-way ANOVA with Tukey's post hoc test. Scale bars in (**C**, **F-H**) represent 50 μm.

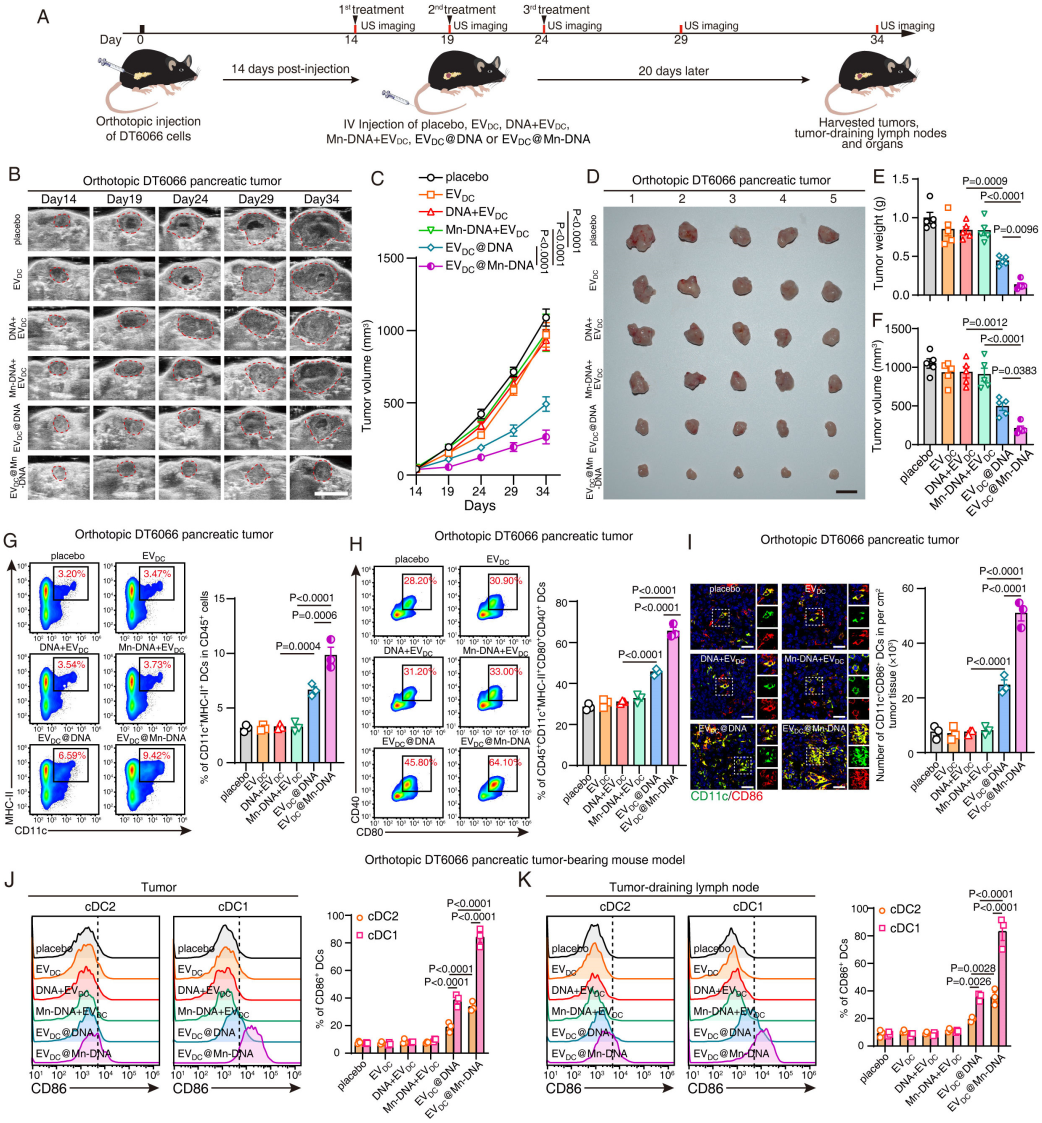

Figure S6. EV$_{DC}$@Mn-DNA treatment suppresses orthotopic DT6066 pancreatic tumor growth and activates DCs in tumors and lymph nodes. (**A**) Schematic of the treatment regimen and ultrasound (US) imaging schedule for mice bearing orthotopic

DT6066 pancreatic tumors. (**B**) Representative ultrasound images showing tumor growth over time for each treatment group. Red line indicates tumor position. (**C**) Tumor growth curves in each group (n = 5 mice per group). (**D**) Representative gross tumor images from each group. (**E**, **F**) Final tumor weight and volume for each group (n = 5 mice per group). (**G**) FACS analysis of CD11c and MHC-II expression in $CD45^+$ DCs from orthotopic DT6066 tumors. Bar chart shows the percentage of $CD11c^+MHC\text{-}II^+$ DCs within the total $CD45^+$ DC population (n = 3 mice per group). (**H**) FACS analysis of CD40 and CD80 expression in $CD45^+CD11c^+MHC\text{-}II^+$ DCs from tumors. Bar chart shows the percentage of $CD45^+CD11c^+MHC\text{-}II^+CD80^+CD40^+$ DCs in the total $CD45^+CD11c^+MHC\text{-}II^+$ DC population (n = 3 mice per group). (**I**) Representative images of CD86 and CD11c co-immunostaining on tumor sections. Bar chart quantifies $CD11c^+CD86^+$ DCs ($\times 10^3/cm^2$) in each group (n = 3 mice per group). (**J**, **K**) FACS analysis quantified $CD86^+$ cells within cDC1 and cDC2 subsets in tumors and tumor-draining lymph nodes following treatment. Bar charts summarize the percentage of $CD86^+$ DCs within each subset across the different experimental groups. Results represent mean ± S.E.M. (**C**, **J**, **K**) Two-way ANOVA with Tukey's post hoc test. (**E-I**) One-way ANOVA with Tukey's post hoc test. Scale bars in (**B**, **D**) represent 1 cm. (**I**) 50 μm.

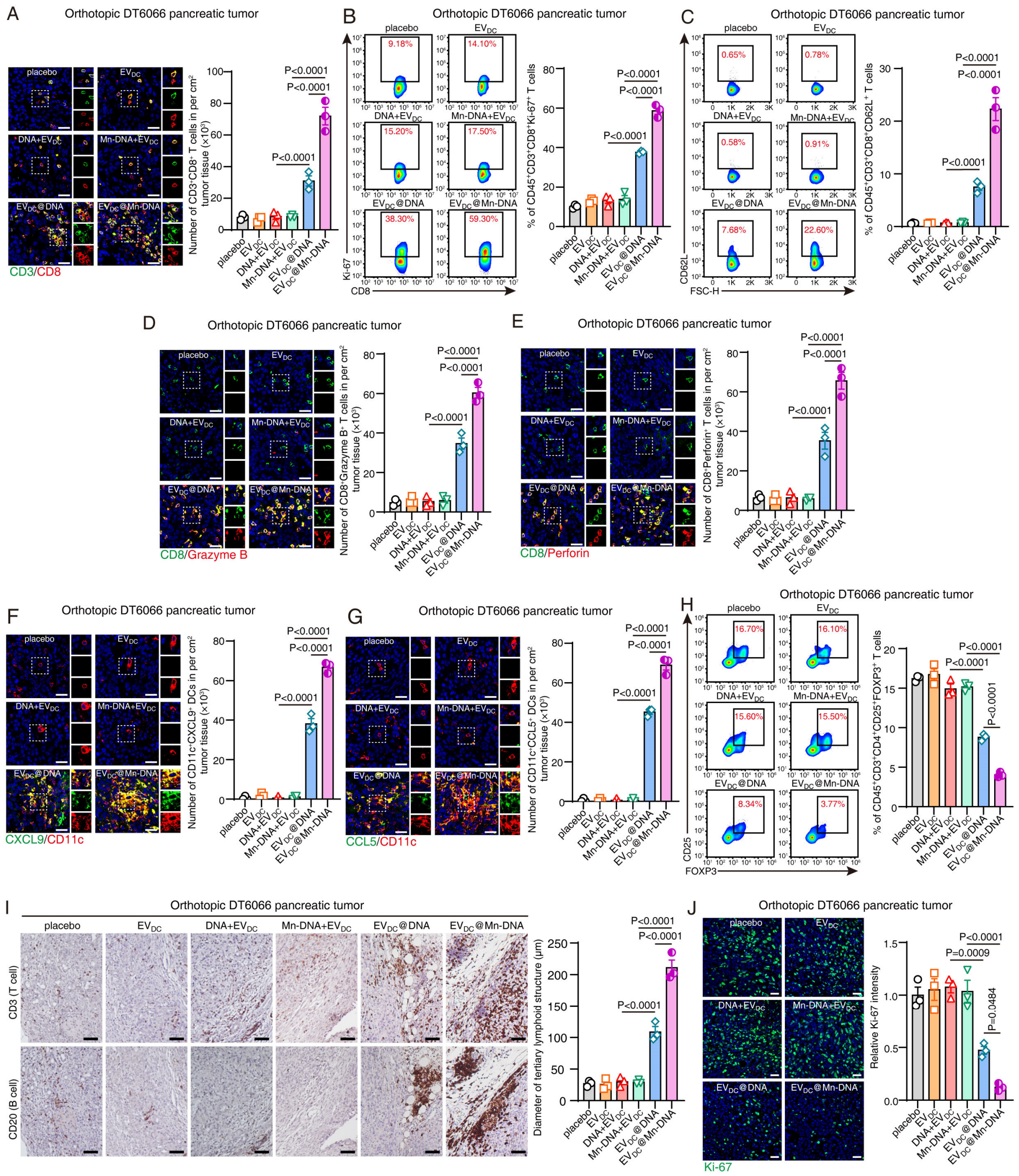

Figure S7. EV_{DC}@Mn-DNA treatment remodels the tumor immune microenvironment in an orthotopic DT6066 tumor–bearing mouse model. (A) Representative images of CD3 and CD8 co-immunostaining on tumor sections from each group. Bar chart shows the number of $CD3^+CD8^+$ T cells ($\times 10^3/cm^2$) per group (n = 3 mice per group). (**B**, **C**) FACS analysis of proliferation marker Ki-67 and central memory marker CD62L in $CD45^+CD3^+CD8^+$ T cells from tumors. Bar charts show the

percentages of CD45⁺CD3⁺CD8⁺Ki-67⁺ or CD45⁺CD3⁺CD8⁺CD62L⁺ cells within the total CD45⁺CD3⁺CD8⁺ T cell population (n = 3 mice per group). (**D**, **E**) Representative images of Granzyme B/Perforin co-staining with CD8 on tumor sections from each group. Bar charts show the number of CD8⁺Granzyme B⁺ or CD8⁺Perforin⁺ T cells ($\times 10^3/cm^2$) per group (n = 3 mice per group). (**F**, **G**) Representative images of CXCL9 or CCL5 co-staining with CD11c on tumor sections. Bar charts show the number of CD11c⁺CXCL9⁺ or CD11c⁺CCL5⁺ DCs ($\times 10^3/cm^2$) per group (n = 3 mice per group). (**H**) FACS analysis of regulatory T cell markers CD25 and FOXP3 in CD45⁺CD3⁺CD4⁺ T cells from tumors. Bar chart shows the percentage of CD45⁺CD3⁺CD4⁺CD25⁺FOXP3⁺ T cells within the total CD45⁺CD3⁺CD4⁺ population (n = 3 mice per group). (**I**) Representative immunohistochemical images of CD3/CD20 on serial tumor sections. Bar chart shows the diameter of tertiary lymphoid structures per group (n = 3 mice per group). (**J**) Representative images of Ki-67 immunostaining on tumor sections. Bar chart shows relative Ki-67 intensity for each group (n = 3 mice per group). Results represent mean ± S.E.M. (**A-J**) One-way ANOVA with Tukey's post hoc test. Scale bars in (**A**, **D-G**, **I**, **J**) represent 50 μm.

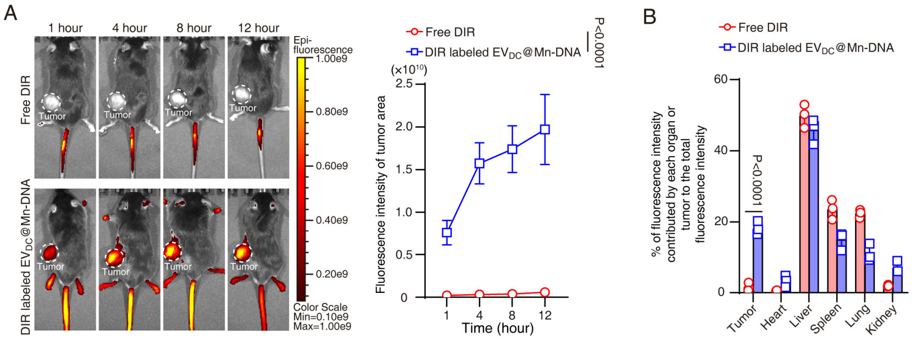

Figure S8. EV$_{DC}$@Mn-DNA efficiently accumulates in tumor tissue. (**A**) Infrared

fluorescence imaging of DT6066 subcutaneous tumor–bearing mice after intravenous injection of either DIR dye alone or DIR-labeled EV_{DC}@Mn-DNA. Representative in vivo images over time are shown for each group, with dashed lines indicating tumor locations. Line graph depicting infrared fluorescence intensity within the tumor region over time for each group. (**B**) Bar chart showing the distribution of DIR dye alone or DIR-labeled EV_{DC}@Mn-DNA in tumors and major organs 12 hours after injection (n = 3 mice per group). Results represent mean ± S.E.M. (**C**, **D**) Two-way ANOVA with Tukey's post hoc test.

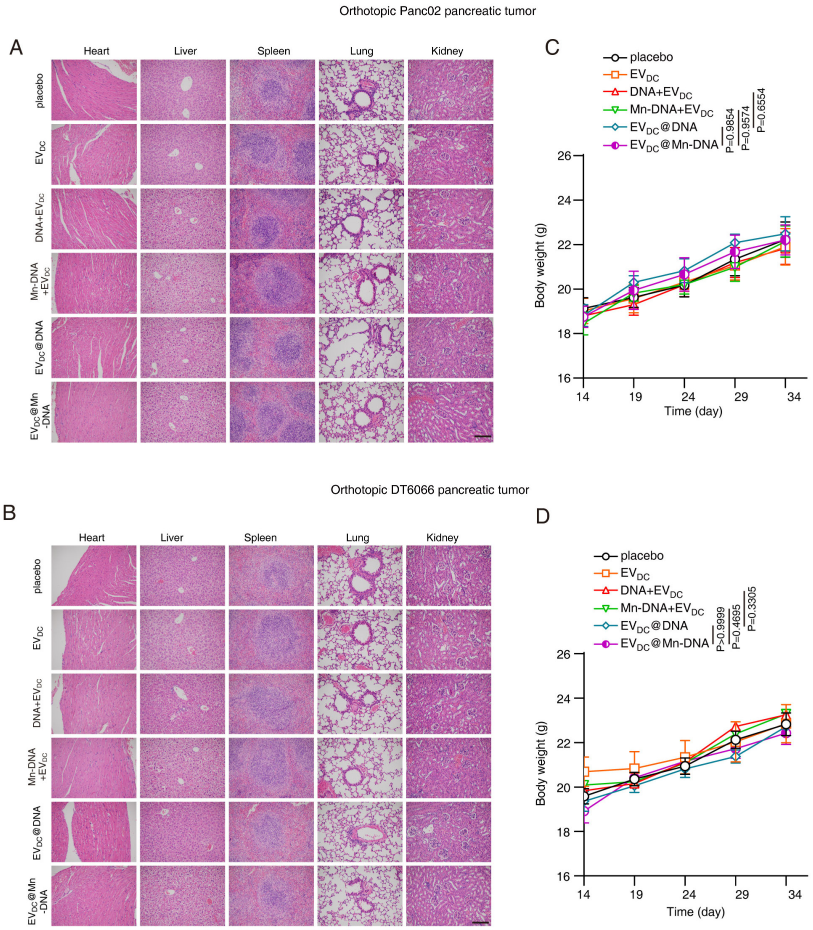

Figure S9. EV_{DC}@Mn-DNA treatment shows no apparent side effects on major organs or body weight. (**A**, **B**) Representative H&E-stained images of major organs

from each treatment group in orthotopic Panc02 or DT6066 pancreatic tumor–bearing mice. (**C**, **D**) Body weight of mice in each group monitored throughout the treatment period (n = 5 mice per group). Results represent mean ± S.E.M. (**C**, **D**) Two-way ANOVA with Tukey's post hoc test. Scale bars in (**A**, **B**) represent 50 µm.

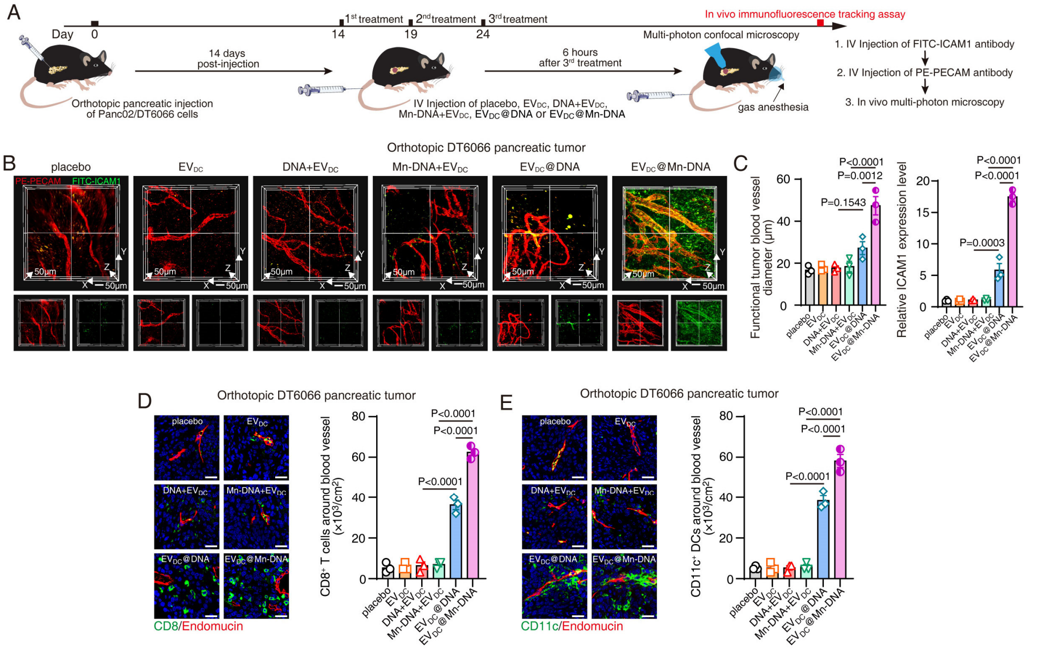

Figure S10. Uptake of EV$_{DC}$@Mn-DNA by endogenous DCs restores vascular-immune balance, promoting immune cell extravasation. (**A**) Diagram represents the in vivo multiphoton confocal imaging of blood vessel function (PE-PECAM antibody perfused blood vessels) and endothelial cell-ICAM1 expression (FITC-ICAM1 stained blood vessels) in orthotopic pancreatic tumors after treated with the indicated treatments. (**B**) Representative 3D images from in vivo multiphoton confocal imaging of orthotopic DT6066 pancreatic tumors for each treatment group. Split images are shown below. (**C**) Bar charts showing functional blood vessel diameters (left) and relative ICAM1 expression levels (right) in each group (n = 3 mice per group). (**D**)

Representative co-immunostaining images of endomucin and CD8 on tumor sections from each group. Bar chart quantifies $CD8^+$ T cells ($\times 10^3/cm^2$) surrounding blood vessels. (**E**) Representative co-immunostaining images of endomucin and CD11c on tumor sections from each group. Bar chart quantifies $CD11c^+$ DCs ($\times 10^3/cm^2$) surrounding blood vessels. Results represent mean ± S.E.M. (**C**-**E**) One-way ANOVA with Tukey's post hoc test. Scale bars in (**B**, **D**, **E**) represent 50 µm.

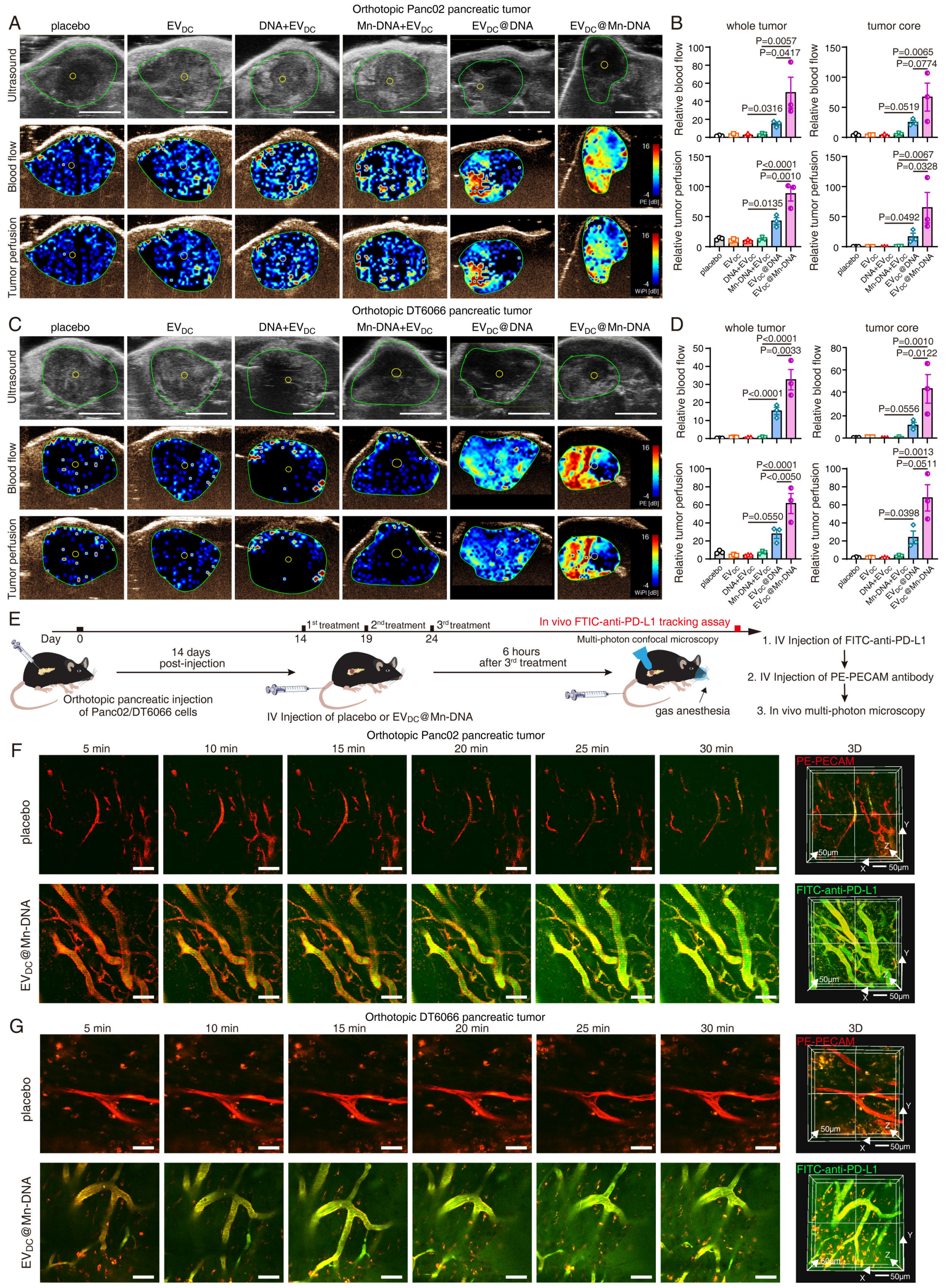

Figure S11. EV_{DC}@Mn-DNA treatment induces tumor vasculature remodeling and promote drug delivery. (**A-D**) Representative microbubble contrast ultrasound images of orthotopic Panc02 and DT6066 pancreatic tumors after treatments are shown.

Bar charts show relative tumor blood flow (top) and perfusion (bottom) for the entire tumor (left) and tumor core (right) in each group (n = 3 mice per group). (**E**) Schematic diagram outlining the in vivo anti-PD-L1 delivery tracking experiment in mice bearing orthotopic Panc02/DT6066 pancreatic tumors after treated with placebo or EV_{DC}@Mn-DNA. (**F**, **G**) Exemplary images of in vivo multi-photon confocal imaging depicting FITC-conjugated anti-PD-L1 distribution in orthotopic Panc02/DT6066 pancreatic tumors from each group over time. Three-dimensional (3D) images at the endpoint are also provided. Scale bars in (**A**, **C**) represent 1 cm. (**F**, **G**) 50 μm. Results represent mean ± S.E.M. (**B**, **D**) One-way ANOVA with Tukey's post hoc test.

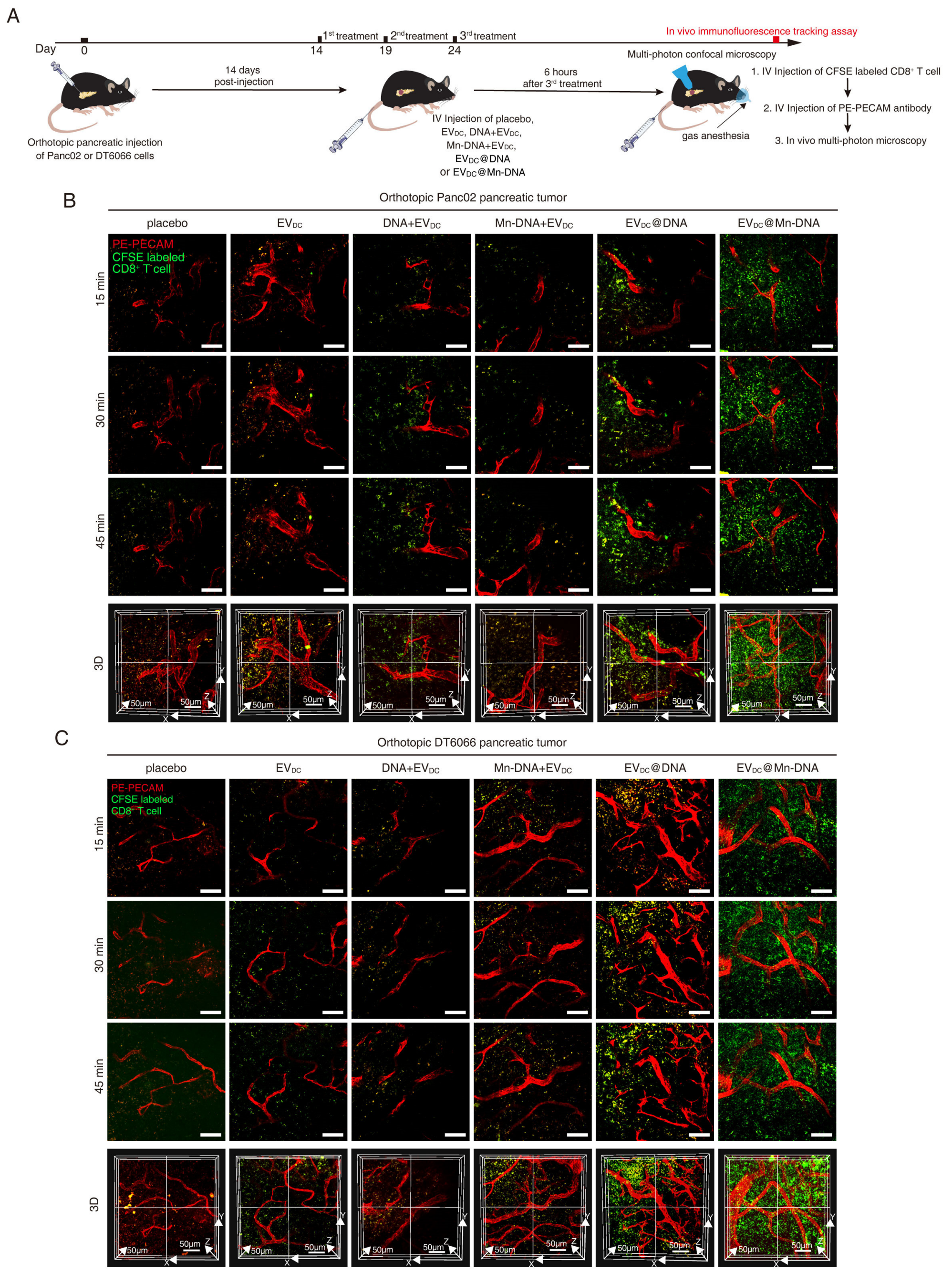

Figure S12. EV_{DC}@Mn-DNA treatment increases $CD8^+$ T cell infiltration in orthotopic pancreatic tumors. (**A**) Diagram showing the in vivo CFSE fluorescently labeled $CD8^+$ T cell tracking experiment in placebo or EV_{DC}@Mn-DNA treated mice

bearing orthotopic Panc02/DT6066 pancreatic tumors. (**B**, **C**) Illustrative images of in vivo multi-photon confocal imaging displaying CFSE fluorescently labeled CD8$^+$ T cell distribution in orthotopic Panc02/DT6066 pancreatic tumors from each group over time. Three-dimensional (3D) images at the endpoint are also included. Scale bars in (**B**, **C**) represent 50 μm.

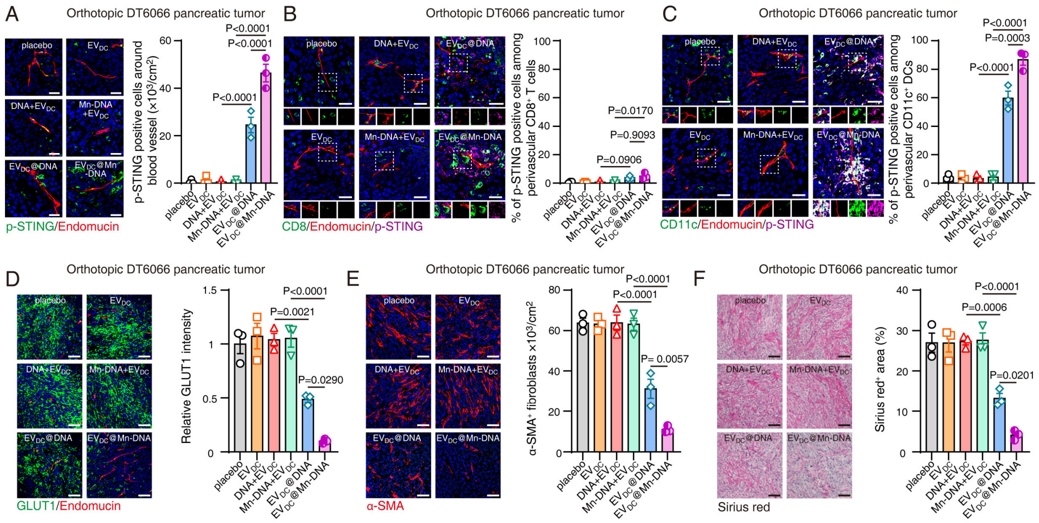

Figure S13. EV$_{DC}$@Mn-DNA treatment remodels the tumor microenvironment in mice bearing orthotopic DT6066 pancreatic tumors. (**A**) Representative images of endomucin and p-STING co-immunostaining on tumor sections from each group. Bar chart quantifies p-STING$^+$ cells (×10^3/cm^2) surrounding blood vessels in each group. (**B**, **C**) Representative tumor sections from each treatment group stained for endomucin, p-STING, and CD8/CD11c are shown. Quantification indicates the proportion of perivascular CD8$^+$ T cells or CD11c$^+$ DCs expressing p-STING in each group. (**D**) Tumor sections stained for endomucin and GLUT1 are presented, with bar graphs displaying relative GLUT1 signal intensity (n = 3 mice per group). (**E**) α-SMA immunostaining images of tumor sections are shown, and bar graphs depict the density

of α-SMA^{+} fibroblasts ($\times 10^3$/cm^2) in each group (n = 3 mice per group). (**F**) Sirius red-stained tumor sections are shown, with bar graphs indicating the percentage of Sirius red^{+} fibrotic area (n = 3 mice per group). Results represent mean ± S.E.M. (**A**-**F**) One-way ANOVA with Tukey's post hoc test. Scale bars in (**A**-**F**) represent 50 μm.

Table S1. The sequences of RT-PCR primers used in this study.

Primer name	Sequence (5'-3')
m- GAPDH-F	TGACCTCAACTACATGGTCTACA
m- GAPDH-R	CTTCCCATTCTCGGCCTTG
h-ACTB-F	CATGTACGTTGCTATCCAGGC
h-ACTB-R	CTCCTTAATGTCACGCACGAT
h-CXCL11-F	GACGCTGTCTTTGCATAGGC
h-CXCL11-R	GGATTTAGGCATCGTTGTCCTTT
h-CCL5-F	CCAGCAGTCGTCTTTGTCAC
h-CCL5-R	CTCTGGGTTGGCACACACTT
h-IL12A-F	CCTTGCACTTCTGAAGAGATTGA
h-IL12A-R	ACAGGGCCATCATAAAAGAGGT
h-IFNB1-F	ATGACCAACAAGTGTCTCCTCC
h-IFNB1-R	GGAATCCAAGCAAGTTGTAGCTC
h-ICAM1-F	ATGCCCAGACATCTGTGTCC
h-ICAM1-R	GGGGTCTCTATGCCCAACAA
h-VCAM1-F	GGGAAGATGGTCGTGATCCTT
h-VCAM1-R	TCTGGGGTGGTCTCGATTTTA
h-ANGPT4-F	CAAGAAAGGGCTAACGCCTC
h-ANGPT4-R	GGCTTCGTTGCATTGGACA
m-ACTB-F	GGCTGTATTCCCCTCCATCG
m-ACTB-R	CCAGTTGGTAACAATGCCATGT
m-IFNb1-F	CTGGCTTCCATCATGAACAA
m-IFNb1-R	AGAGGGCTGTGGTGGAGAA
m-CCL5-F	ACCTTCTCCTGCGGGAATCCAA
m-CCL5-R	TTCTCTGGGTTGGCACACAC
m-IFIT1-F	CAAGGCAGGTTTCTGAGGAG
m-IFIT1-R	GACCTGGTCACCATCAGCAT
m-ISG15-F	CTAGAGCTAGAGCCTGCAG
m-ISG15-R	AGTTAGTCACGGACACCAG
m-CD86-F	CATGGGCTTGGCAATCCTTA
m-CD86-R	AAATGGGCACGGCAGATATG
m-CXCL11-F	GACGCTGTCTTTGCATAGGC

m-CXCL11-R	GGATTTAGGCATCGTTGTCCTTT
m-CXCL12-F	TGCATCAGTGACGGTAAACCA
m-CXCL12-R	TGCACACTTGTCTGTTGTTGTT
m-CCR7-F	TGTACGAGTCGGTGTGCTTC
m-CCR7-R	GGTAGGTATCCGTCATGGTCTTG

m= mouse, h= human
